# Terrorist attacks and bank financial stability: evidence from MENA economies

**DOI:** 10.1007/s11156-022-01043-1

**Published:** 2022-02-19

**Authors:** Marwa Elnahass, Mohamed Marie, Mohammed Elgammal

**Affiliations:** 1 Accounting and Finance, Newcastle University Business School, Newcastle University, London, UK; 2 Faculty of Commerce, Cairo University, Giza, Egypt; 3 College of Business and Economics, Qatar University, Doha, Qatar

**Keywords:** Terrorism, Financial stability, Bank risk, Financial performance, C23, G01, G21, G28, L50, M4

## Abstract

This study examines the impact of terrorism on bank stability, represented by bank risk and financial performance. We consider banks from 14 countries located in the Middle East and North Africa region for the period 2010–2018 using both the three-stage least-square and the generalised method of moments. The results provide strong evidence that banks located in countries with high exposure to terrorist attack exhibit low financial stability, due to high bank risk (i.e., high credit and insolvency risk). However, these banks show high financial performance (i.e., high profitability and cost efficiency), on average. Our results also show differential impacts on bank stability for countries marked as more (less) exposed to risk of attacks. For banks located in high-income-generating countries, we find that exposure to terrorism is associated with low financial performance and high credit risk, which is the opposite case for low-income-generating countries. Our results also indicate high systemic risk for listed banks operating under high terrorism risk exposure.

**Supplementary Information:**

The online version contains supplementary material available at 10.1007/s11156-022-01043-1.

## Introduction

As the banking industry moves towards sophisticated financial technology, evidence relating to the financial stability of banks is still accumulating. Banks play an important role in economic growth, through their role as intermediaries between savers and depositors. They also provide funds for activities that support enterprise, which fosters a strong and healthy economy. Bank financial stability is a subject of considerable interest in financial services and economics, especially after the financial crisis of 2007. Financial instability can be very costly for the banking industry due to its contagion or spillover effects to other parts of the economy. Hence, it is fundamental to have a sound, stable and healthy financial system to support the efficient allocation of resources and distribution of risks across the economy. Corporate risk-taking, monitoring of risks, and promoting financial performance and resilience in the global banking sector remain a central focus for banks’ directors/managers, international regulators and investors (Elnahass et al. 2021).

While the majority of prior studies focused on operational, financial, macroeconomic and microeconomic determinants of banking financial stability (e.g., Chan and Milne 2014; Ashraf and Rizwan 2016; Rumler and Waschiczek 2016; Elnahass et al. 2020a, b, 2021; Trinh et al. 2020a), limited attention has been paid to identifying the role of political stability in prompting bank stability. Accordingly, it remains questionable whether banks can still operate and mitigate risk during accelerating political events like terrorism,^1^ particularly for those banks located in vulnerable countries. In a joint survey by the Centre for the Study of Financial Innovation (CSFI) and PricewaterhouseCoopers (PwC) in 2010, ‘political instability’, along with its implications, was rated as the most important risk (Liu and Ngo 2014). According to Calomiris and Haber (2014), politics remain an important driver behind the frequent and systematic banking crises. Terrorist attacks are marked as the most detrimental political event for economies and societies.

Terrorism has several economic consequences, which can be segmented into short-term direct effects, medium-term confidence effects and longer-term productivity effects. The economic costs of terrorism encompass the demolition of life and property, reestablishment of the systems and the infrastructure affected, and the provision of temporary living assistance. Terrorism affects the economy of the country by diverting foreign direct investment (FDI), disturbing financial markets, slowing domestic investments, and even affecting the consumption plans and government spending (Abadie and Gardeazabal 2008; Sandler and Enders 2008). The financial and economic decisions are highly impacted by the psychological terror caused during the terrorism events. These events decrease investor confidence and produce a wide macroeconomic shock. Eventually, terrorism restricts economic growth and amplifies unemployment and poverty problems, which consequently affect the resilience of the financial sector (see Chesney et al. 2011; Younas 2015; Arif and Suleman 2017; El Ouadghiri and Peillex 2018).

Previous studies have largely focused on economic stability and drivers of terrorism. Some have generally documented the presence of an association between terrorism and economic activities (De Mesquita 2005; Bardwell and Iqbal 2021), whilst others indicate that economic downturns are associated with high numbers of terrorist attacks (Blomberg et al. 2004; Drakos and Gofas 2004; Efobi et al. 2015). On the other hand, bank financial stability literature has commonly investigated issues related to corporate governance, risk monitoring and financial performance and, recently, emerging questions related to the COVID-19 pandemic (e.g., Chan and Milne 2014; Rumler and Waschiczek 2016; Elnahass et al. 2020a, b, 2021; Trinh et al. 2020a). This literature is generally classified into three categories: one strand focuses on the determinants of financial performance, the second strand investigates the determinants of banks risk, and a few studies investigate factors that affect both bank performance and risk in relation to the adopted governance and internal control mechanisms. However, no study to the best of our knowledge has empirically assessed the impact of terrorism on bank financial stability, in either developing or developed countries. Accordingly, this study aims to cover the existing gap in the banking and finance literature through presenting comprehensive and systematic analyses for the impact of a major political risk like terrorism on bank financial stability.

In line with Caprio and Honohan (1999) and Belghitar et al. (2019), we assume that political instability within developing countries makes them more vulnerable to financial stress and promotes bank financial instability. This is the fundamental premise investigated in our study. An empirical assessment for countries with more frequent terrorism incidents offers an interesting setting because incidents of terrorist attacks are likely to turn out to be a routine element (systematic risk) over time for many economies sensitive to these attacks. High frequency of attacks would reflect an erosion of sensitivity to prolonged terrorism, in other words ‘normalisation of terror’ (Arin et al. 2008; Peleg et al. 2011). Furthermore, prior studies (e.g., Chesney et al. 2011; Kollias et al. 2011) show that systematic implications of terrorism are not only restricted to stock markets but can also be observed in other financial markets including bonds, government securities, the repo market and commodity markets.

We utilise unique data for banks located within the Middle East and North Africa (MENA) region. Because of excessive and congested regional conflicts, political violence and vast oil wealth, MENA plays a key role in the prospects for global terrorism, world tranquillity and economic prosperity (Kim and Sandler 2020). Therefore, the choice of MENA countries in our study is justified by the large number of terrorist attacks over the last two decades (Bardwell and Iqbal 2021). According to the Global Terrorism Index (2019), between 2002 and 2018, the MENA region recorded the highest number of fatalities due to terrorism (93,700, representing 42% of the global total). In fact, the MENA region is constantly suffering from recurring terrorist attacks in addition to political unrest, which reflects the importance of understanding the impact of terrorism on bank stability in this region (Kim and Sandler 2020).^2^

We employ different risk indicators (i.e., insolvency, credit, liquidity, asset and operational risks) and performance measures (i.e., profitability ratio and cost to income ratio). We measure terrorism risk utilising the Global Terrorism Index (GTI). The analysis is based on a sample of 954 bank-year observations (106 banks) in 14 countries for the period from 2010 to 2018 using both 3SLS and GMM models. Our findings suggest that banks with high terrorism exposure exhibit an overall high-risk profile, and hence have low bank stability. These banks report significantly high credit risk and high insolvency risk. The economic consequences of terrorism lead to substantial economic deterioration, which adversely affects bank stability. Moreover, we find that the risk of terrorism is associated with low liquidity risk for banks, which can be explained by the substantial bailouts offered by the governments to help their economies to overcome the terrifying consequences. However, our results show that a high risk of terrorist attacks is associated with better financial performance (i.e., high profitability and better cost efficiency). These results are consistent with the trade-off hypothesis between risk and return. The increase in the risk exposure of banks because of terrorism will drive banks to maintain high profit margins as compensation for the increased risk. In addition, such improvement in the profitability position of banks can be explained by the impact of economic stimulation packages as well as cuts in the interest rate alongside liquidity injections by institutions and governments during/after the terrorist attacks. Altogether, these factors may support the recovery of the financial system through increasing the demand on bank services for restructuring plans from one side and decrease the main element of bank cost, interest, from the other side.

We perform additional tests to identify the differential effects of terrorism on bank stability among countries that are characterised by either high or low exposure to terrorist attacks. We find that the impact of terrorism on bank stability is different across these two categories of countries. Terrorism increases the insolvency risk for banks located in countries with a high risk of attacks; however, these banks demonstrate better financial performance. In contrast, banks within countries at low risk of terrorist attacks report higher credit risk and lower financial performance in terms of cost efficiency and profitability. We additionally investigate the impact of systematic risk for our sampled banks, using CoVaR analyses; we find high systemic risk for listed banks operating under high terrorism risk exposure. Terrorist attacks increase the volatility of financial markets, which leads to high risk-taking and low financial stability for MENA banks.^3^ We further investigate the terrorism effect among high-income and low-income-generating countries and the bank’s life cycle. We also employ alternative estimation procedures and models. Our findings remain consistent with our predictions and confirm the main results.

This study makes several contributions to the literature on banking and financial markets. First, this is the first study that employs a unique dataset for MENA countries to investigate the impact of terrorism on bank stability. Such absence of up-to-date empirical analyses for MENA is quite surprising given that this region has been the birthplace for many notorious terrorist groups (e.g., al-Qaida in Iraq, al-Nusra, al-Qaida in the Arabian Peninsula and Islamic State [IS]) and has been embroiled in conflicts over the last decades. We employ several bank risk and financial performance measures and offer systematic and joint tests for this unique study setting. Hence, our work contributes to the growing banking studies which concentrate on measuring economic stability and risk mitigation, but which have not captured the individual and joint effects of terrorist attacks on financial performance and bank risk (e.g., Gasbarro et al. 2002; Imerman 2020; Trinh et al. 2020b; Elnahass et al. 2021). In fact, our results (e.g., highlighting the damaging effect of terrorism on bank risk) extend earlier work on the economic impacts of terrorism (Estrada et al. 2015; Asongu and Nwachukwu 2017a, b; Kim and Sandler 2020). This study is also of relevance to investment choices in the emerging banking industry in general (e.g., Rahman and Hassan 2013; Fosu et al. 2020), and studies examining financial determinants of bank stability and corporate policies in monarchies in particular (Choi 2010). We highlight the differential effects of terrorism on the bank stability within countries subject to high versus low risk of attacks and those classified as high/low income-generating countries. Moreover, we add to the sizable literature which examines the effect of terrorism on the overall stability of financial markets (Eldor and Melnick 2004; Graham and Ramiah 2012; Su et al. 2019).

Our results offer important insights to policymakers, emphasising that terrorism represents one of the key systematic risks, which must be constantly reviewed and measured in vulnerable economies as well as aligned with existing/new measures developed for the global banking industry. While the macro-effect symptoms of terrorism are becoming increasingly visible in MENA countries, with a higher attack frequency rate than other regions in the world, the impact of terrorism on banking stability can be commonly observed across our sampled countries. The results of this study can inform both investors’ investment choices and regulators, regarding the distinct impacts of terrorism on the volatility of capital markets and risk-taking in banks. By identifying and highlighting the adverse impact of terrorism on bank stability in our study, counter-terrorism strategies should consider developing, *ex nate*, protective measures/ guidance for banks to mitigate the devastating effects that extremist groups could inflict on MENA banks’ stability, with an expected transmitted impact to the global banking industry. Our findings should elicit immediate responses from regulators and policymakers, pressing them to draw up action plans to set up banking unions among affiliated regions. Such unions are essential requisites to mitigate different types of financial risks that could be presented by an evolving idiosyncratic crisis in the near future, which, consequently, could influence financial stability in vulnerable economies and beyond. Our study has revealed the signals of enhanced financial performance for banks located in economies which high risk exposure. Such high financial performance is likely to represent a trade-off (i.e., compensation) for increased insolvency risk. Banks operating within low risk exposure countries tend to have some difficulties in generating enough income, acquiring governmental financial aid, and/or utilise their financial assets and capital to absorb this political shock. We argued that, even though financial aid and bailouts are offered to support banks in some countries, the financial impact of terrorism on the MENA banking industry would be felt for a long time to come. This study calls for coordinated responses among policymakers to protect and support the banking industry (e.g., via conflict aid and anti-terrorism resource pre-positioning) during episodes of unprecedented political events. International regulators need to develop robust solvency and stress-testing frameworks for banks located in emerging economies in order to identify, assess, monitor and control material risks resulting from terrorism incidents.

The next section presents the literature review and background. Section 3 outlines the hypotheses. Section 4 presents the data. Section 5 outlines the methodology and measures. Sections 6 and 7 report empirical results and additional tests. Section 8 concludes the study.

## Background

### Bank stability and terrorism

In the short term, there are some severe direct and indirect costs associated with terrorist attacks, which are related to substantial economic repercussions by destroying business and consumer confidence, which reduce both consumption and investment, which in turn hinders economic growth (Estrada et al. 2015). Terrorist attacks increase mobilisation from the attacked area, which affects economic activities and demand for financial services. In the long term, terrorism may affect the economy’s productivity by raising the costs of transactions through increased security measures, higher insurance premiums, higher financial costs, and other counter-terrorism regulations (Johnston and Nedelesc 2006). The terrorism literature documents a positive association between economic depressions and terrorism (Blomberg et al. 2004; Drakos and Gofas 2004; De Mesquita 2005; Bardwell and Iqbal 2021). Moreover, terrorism creates severe threats for life and for economic losses, which affect economic prosperity and governance (see Abadie and Gardeazabal 2003; Frey 2004; and Asongu and Nwachukwu 2017a, b). Furthermore, Shahbaz (2013) finds that terrorism increases inflation, while Raza and Jawaid (2013) show that an increase in terrorism events reduces tourism revenues. Estrada et al. (2015) and Sandler and Enders (2008) explain the detrimental impact of terrorism on the economy by the rational choice theory because the government’s decision to fight terrorism should be a trade-off between the costs of acquiescing to the terrorism agenda and the costs of fighting it.

The innate suddenness of terrorism events creates fear in the financial markets, which affects investors’ confidence and causes financial market instability (Eldor and Melnick, 2004; Shahbaz et al. 2013). Financial instability can be very costly for the banking industry due to its contagion or spill over effects to other parts of the economy. Hence, it is fundamental to have a sound, stable and healthy financial system to support the efficient allocation of resources and distribution of risks across the economy during incidents of terrorism. During and after terrorist attacks, the economic output and savings will diminish accompanied with trade losses and higher insurance and risk premiums and interest rates with an increase in capital flight (Sandler and Enders 2008; Younas 2015; Efobi and Asongu 2016). According to Bandyopadhyay et al. (2014), the economic consequences of terrorism will reduce both domestic and foreign investment. Hence, banking operations and performance are likely to be affected because they may face the dilemmas of shortage in financial resources, an increase in the demand for such resources for restoring development, and increasing investment fears and uncertainty.

Prior studies have studied bank stability in line with several determinants such as bank risk (Bitar et al. 2018; Ding and Sickles 2018; Ozili 2019a), profitability (Shrieves and Dahl 1992; Lee and Hsieh 2013; Ozili 2019b), liquidity (Kim and Sohn 2017; Ding and Sickles 2018; Jiang et al. 2020), bank ownership (Boateng et al. 2015; Ozili 2018), market share and firm valuations (Jiang et al. 2020; Elnahass et al. 2020a). The second set of bank stability literature draws more attention to the impact of corporate governance on bank stability and performance (see Elyasiani and Zhang 2015; Faleye and Krishnan 2017; Kress 2018; Abdelsalam et al. 2020; Elnahass et al. 2020b, 2021; Trinh et al. 2020a). Finally, other studies show that bank stability can be affected by different macroeconomic factors, i.e., fluctuation in economic cycles (Segoviano and Goodhart 2009), real output growth (Jokipii and Monnin 2013), unemployment rate (Heffernan and Fu 2008) and GDP (Boateng et al. 2015). However, the impact of such economic variables on bank stability varies from one country to another (Segoviano and Goodhart 2009). According to Howarth and Quaglia (2013), there was an association between political economy characteristics and new capital requirements in the European Union after the financial crisis (2007–2009). Tamadonejad et al. (2016) find that political and economic factors can predict banking crises. Furthermore, Rajhi and Albuquerque (2017) document that both natural disasters and state fragility determine bank stability. Ashraf (2017) shows a positive relationship between political institutions’ soundness and bank risk-taking, highlighting that better political institutions stimulate credit market competition and generate moral hazard problems that lead banks to increase risk-taking.

In summary, no prior literature has examined the effect of terrorism on bank financial performance and bank risk, jointly. The variation in the findings of previous research with respect to the impact of terrorism on the financial markets invites us to consider bank characteristics (i.e., bank risk-taking behaviour and financial performance) while considering variation in risk exposure to terrorism for highly vulnerable economies.

### Terrorism in the MENA region

Terrorism has been constantly linked to developing economies with low economic stability (De Mesquita 2005; Estrada et al. 2015), high rate of unemployment, and limited opportunities for people to upgrade their economic and social class (Muller and Seligson 1987). The effects of terrorism seem to differ from developed to developing countries, according to the characteristics of the country in which the incident has occurred. Countries with low levels of economic growth are expected to be highly sensitive to terrorism compared with countries that have higher initial economic growth rates, although firms operating in wealthier or more democratic countries face greater volatility in stock returns relative to firms operating in developing countries (Johnston and Nedelesc 2006; Drakos 2010). Arif and Suleman (2017) find that the impact of prolonged terrorism on stock prices has different signs across market sectors. An example from the developing countries is that the 25.53% drop in the Indonesian stock market persisted for five days after the terrorist attack on Bali Island in 2002 (Ramiah and Graham 2013). In developed economies, a popular example for the impact of terrorism on economy and stock markets is the closing of the New York Stock Exchange (NYSE) for one week after the 9/11 attack, followed by a 14% fall in the Dow Jones index (Lenain et al. 2002).

Since 1989, the MENA region has been plagued by growing terrorist attacks and conflicts, indicating substantial increases in the region’s military expenditure (Sandler and George 2016; Stockholm International Peace Research Institute, 2019). For instance, MENA’s global share of terrorist attacks rose from 9.8% during 1970–1989 to 36.1% during 2002–2018. Although the region has witnessed significant economic growth rates which have opened the door for the growing banking industry, the resilience in Middle Eastern autocracies has been exhaustively exposing the MENA region to many conflicts and political terrors in the last 10 years since the Arab Spring started in 2010 (Kim and Sandler 2020). The spill over of terrorism to Europe and North America, coupled with the recent refugee exigency in Europe stemming from three civil wars in the MENA region, illustrate the region’s impact on political stability and violence (Kim and Sandler, 2020). Moreover, the MENA countries showed the largest increase in terrorist impacts in 2018 and suffered the highest economic loss of $12.2 billion due to terrorism in that year (37% from the global economic impact of terrorism).^4^

Several MENA countries are controlled by non-democratic regimes, which makes them more vulnerable to terrorism because of weak law enforcement and politicians’ corruption (Choi 2010; Bardwell and Iqbal 2021). In terms of economic structures and socio-economic development, MENA countries form a highly diverse group. The oil-rich countries of the Gulf Cooperation Council (GCC) have very high per capita income levels, relatively low levels of economic diversification, and ample financial resources available for social service provision. Other MENA countries have per capita income ranging between lower-middle and upper-middle income countries and contain the majority of the population in the region. These countries represent relatively more diversified economies (Karshenas et al. 2014). Moreover, the MENA region has encountered many critical economic challenges. For example, countries that have experienced a revolution are faced with the challenge of rebuilding their economies, whilst other countries in the region have been involved in an oil war. The ongoing Arab Spring still affects the political and economic environments in several MENA countries. This political unrest affects economic stability and increases the vulnerability of financial systems to terrorist attacks. Khandelwal and Roitma (2013) argue that episodes of political instability in the MENA region affect economic development efforts, which are shaped by limited progress in the medium term and a sharp deterioration of macroeconomic variables. Key determinants of domestic and transnational terrorism in the MENA region are represented by the lack of democracy and the post-Arab Spring era (Kim and Sandler 2020). The highest level of terrorism in the 18 years to 2018 in the region was observed in countries that experienced armed conflict (Bardwell and Iqbal 2021).

The economic consequences of terrorist attacks in the MENA region tend to be more damaging when these attacks target, for example, critical economic sectors like oil and gas in GCC countries and tourism in other MENA countries. The key features of the financial infrastructure of many MENA countries are a lack of diversification across resource production sectors, and relatively underdeveloped legal and governance mechanisms for both financial and non-financial firms. Hence, the role of bank credit expansions and contractions in the economy lies firmly on the supply side, rather than as a mechanism for stimulating or contracting demand.

The importance of banks in capital allocation for MENA countries stems from the lack of equity financing and weak domestic stock markets. For some countries in this region, the sovereign seeks to monitor new venture creation and capital allocation, which is best accomplished through the banking sector. For other countries, recent and ongoing political instability is not conducive to an equity-financing framework when contracts and monitoring may be incomplete. Moreover, legal structures are often subject to weak enforcement and oversight and this reduces the willingness of investors to engage in new venture creation via equity capital. As such, MENA banks offer a conduit for capital into the economy in a contiguous fashion. In addition, the war against terrorism would require governments in the affected communities to have dedicated budgets to fight terrorism and bailout packages to rescue and support different economic sectors. Therefore, the consequential economic and financial costs of terrorist attacks are expected to have direct and serious implications on the stability of the banking industry within the MENA region.

## Theoretical framework and hypotheses development

Macroeconomic variables, which are systematic in nature, affect the risk exposure of banks (see Demirgüç-Kunt 1989; Anginer et al. 2014). The political economy theories (Caporaso and Levine 1992) assume that political stability affects different macroeconomic factors and any economic shocks will be transmitted to the economy, affecting the economic activities and risk exposure. Additionally, Brewer and Rivoli (1990) show that political instability as indicated by the frequency of governmental regime, political legitimacy and armed conflict affect perceived country creditworthiness. We also build on the theoretical framework that combines the macroeconomic factors with bank stability. This combination of theories assesses the trade-off between expected return and riskiness of that return, and shows evidence that systematic bank risk is a mix of endogenous and exogenous factors. For example, Demirgüç-Kunt and Detragiache (1998) show that a weak macroeconomic environment (in terms of high inflation and low GDP) is associated with systematic banking sector problems. Moreover, Hughes et al. (2001) and Mester (2008) confirm the link between macroeconomic systematic risk and different measures of bank stability, efficiency and profitability. Additionally, Buch et al. (2014) show that macroeconomic factors including GDP growth, inflation, the Federal Funds rate and house price inflation affect micro-level bank risk. They suggest that expansionary shocks decrease average bank risk and increase average bank lending. The heterogeneity of banks is characterised by idiosyncratic shocks and the asymmetric transmission of common shocks. The nexus between terrorism and bank stability comes through two channels: first, the impact of political stability on macroeconomic factors (Brewer and Rivoli 1990; Caporaso and Levine 1992) and, second, the impact of macroeconomic shocks on bank stability. Terrorist attacks are among the most influential political shocks which threaten the political stability, and, in turn, this will affect economic systematic risk and should increase the risk exposure of banks. According to the trade-off between risk and return theory, if the banks expected risk to increase this should increase their required returns on investment; in other words, banks should seek higher profitability as a compensation for taking more risks.

Terrorism has a detrimental impact on several macroeconomic factors, which, in fact, are expected to lead to negative impacts on bank stability. Terrorist attacks significantly reduce economic growth, financial environments, foreign direct investment (FDI), domestic investments, consumption plans and government spending. Financial and economic decisions are highly impacted by the psychological terror caused by terrorism events. These events decrease investor confidence and produce financial instability. Altogether, this will increase the volatility of financial markets and in turn threaten bank stability (see Kollias et al. 2013; Estrada et al. 2015; Younas 2015; Arif and Suleman 2017; El Ouadghiri and Peillex 2018). According to Larobina and Pate (2009), terrorism affects the business environment by adding additional economic costs. Simser (2011) shows that financial institutions report 12 million filings annually related to combating the financing of terrorism and anti-money laundering suspicious transactions. Involvement in terrorism financing, intended or not, will create serious exposure to many risks, including reputational and operational risks, in addition to prosecution, financial fines and compensation. Johnston and Nedelesc (2006) suggest that financial institutions need to adopt effective contingency planning to mitigate the risks of disruption from terrorist attacks. Financial institutions may face an increasing liquidity risk because of terrorism, e.g., the US government’s securities market and repo market were reduced after 9/11 because the damage to the trading infrastructure, lack of confidence, and the reluctance of market participants to lend out securities, resulted in a lack of money supply (Johnston and Nedelesc 2006). According to the political economy theories (Caporaso and Levine 1992), political stability affects different macrocosmic factors and any economic shocks will be transmitted to the economy, affecting the economic activities and increasing risk exposure. In addition, Brewer and Rivoli (1990) show that political instability affects perceived country creditworthiness. Accordingly, we conjecture that terrorist attacks are among the most important political shocks that threaten the political stability and are likely to promote high economic systematic risk, and hence should increase the risk exposure for banks. This leads to the first hypothesis:

## ***H***_***01***_***: A high exposure to terrorism risk is associated with a high bank risk***

The economic stimulation package, interest rate cuts and liquidity injections after a terrorist attack can help the financial system to recover in the medium and long term. The success of such a stimulation package will vary based on the solidity of the economy and the severity of the terrorist attack. Prior literature provides evidence that terrorism affects the return on banking sector. For example, Liargovas and Repousis (2010) investigate the impact of three terrorist attacks on Greek banking’s stock returns. They report that only the 9/11 attacks had a significant impact on banks’ stock returns because of the US economy’s high interconnection with and impact on the global financial system. Arif and Suleman (2017) report a positive relationship between the terrorist index and financial sector returns in Pakistan. They justify this relationship by highlighting the increasing demand for insurance due to the increasing terrorism risk, from one side. On the other side, the Pakistani banking system has witnessed an increase in deposits and profitability because of the postponing of Pakistan’s foreign debt for 28 years after 9/11 and the return of Pakistanis’ deposits from overseas banks to local banks due to the strict scrutiny of Muslims holding accounts in foreign banks in Western countries. This strict scrutiny may also attract investors from the Middle East to invest in Pakistan than in Europe or America.

Chen and Siems (2004) assume that the stability of the banking sector and its ability to provide adequate liquidity play a vital role in the resilience and speedy recovery of the US capital market from the negative impacts of terrorist attacks. The predicted positive association between the risk of terrorist attacks and bank profitability can be explained through modern portfolio theory, which assumes a trade-off between risk and return.^5^ That is, the increase of the risk exposure of banks because of terrorism will drive banks to require higher profit margins as compensation for the risk (see Demirgüç-Kunt 1989; Anginer et al. 2014). According to the trade-off between risk and return theory, if the banks expected the risk to increase, this should increase their required returns on investment. Banks will seek higher profitability as compensation for taking more risks. In fact, after terrorism events, usually the responsive and rescue packages are based on offering lower interest rates in order to reduce banks’ cost of capital. However, banks usually increase the interest rate margin to compensate for their customers’ increased levels of credit risk during this exogenous shock. These two channels can work together to increase banks’ profitability as the rescue packages which tighten interest rates will reduce banks’ costs; however, this will also increase the demand on bank loans for restructuring to treat the impact of terrorism. However, banks tend to increase the interest rate margin to compensate for the increase in the credit risk of their customers. This leads to the second hypothesis:

## ***H***_***02***_***: A high exposure to terrorism risk is associated with a high bank financial performance***

### Data and sample

The initial sample represents 260 listed and unlisted commercial operating banks in the MENA region during the period 2010–2018. We hand-collected the governance data for board information, ownership structure and audit committee from the banks’ annual reports. In addition, bank-level data is retrieved from Bankscope and DataStream while country-level macroeconomic and governance indicators are retrieved from the World Bank’s World Development Indicators database. We filtered the sample following similar criteria applied in other banking studies (see Mollah et al. 2017; Trinh et al. 2020b). These comprise: (a) banks that have full annual reports available from official websites, published as of the financial year of 31 December; (b) only commercial conventional fully-fledged banks were kept due to poor data availability for Islamic banks within our study’s setting; and (c) banks having full data availability of at least three consecutive years. Our final sample represents balanced panel data of 106 conventional banks (954 bank-year observations) for both listed and unlisted banks in 14 countries during the period 2010–2018. The selection of the sample period avoids the potential effect of the financial crisis period of 2007–2009.

Egyptian banks represent the largest percentage of the sample, forming 20.75% of it, whilst the percentage of United Arab Emirates banks in the sample is 11.32%, but the size of the latter’s assets is often larger than that of the former’s. In addition, the percentage of sample banks from Yemen and Palestine is only 1.7% due to the difficulty of obtaining data for these banks because of the wars and terrorism that these countries are exposed to. However, despite the presence of civil wars and terrorism, Syrian banks have maintained continuous disclosure of data, so their sample was 5.66% of the total.

Table 1 presents the sample distribution by country and bank. The highest concentration of our sample MENA banks is found in Egypt and the United Arab Emirates while Yemen and Palestine reported the lowest bank representation.

**Table 1:** Sample distribution by countries

N	Country	No. banks	Observations	Relative %
1	Bahrain	11	99	10.38
2	Egypt	22	198	20.75
3	Israel	8	72	7.55
4	Jordan	8	72	7.55
5	Kuwait	6	54	5.66
6	Lebanon	6	54	5.66
7	Oman	6	54	5.66
8	Palestine	2	18	1.89
9	Qatar	6	54	5.66
10	Saudi Arabia	8	72	7.55
11	Syria	6	54	5.66
12	Tunisia	4	36	3.77
13	United Arab Emirates	12	108	11.32
14	Yemen	1	9	0.94
Total		106	954	100

The final sample comprises 106 conventional commercial banks (954 observations) in 14 countries for the period from 2010 to 2018

## Methodology and measures

### Measures of bank financial stability

To measure bank financial stability, we employ different indicators for financial performance and bank risk. Firstly, we use three bank risk measures: (i) credit risk, (ii) liquidly risk, and (iii) insolvency risk. Following Abedifar et al. (2013) and Bitar et al. (2017), we measure credit risk using the ratio of loan loss reserves to gross loans (*LLR/GR*). A higher value of *LLR/GR* indicates a higher credit risk for a bank. Our second risk measure is liquidity risk; we measure a bank’s liquidity risk using the ratio of liquid assets to deposits and short-term funding (*LA/DSF*) (Safiullah and Shamsuddin 2018). This is a deposit run-off ratio and shows whether the bank holds more liquid assets to support deposits and short-term funding. The higher the value of this ratio, the lower the bank’s liquidity risk. Finally, we measure insolvency risk by the bank’s ‘*Z-score*’ as a measure of bank probability to default (Rumler and Waschiczek 2016). The *Z-score* has been calculated as the sum of return on assets and capital assets ratio, scaled by the standard deviation of return on assets (Lee and Hsieh 2013). A high *Z-score* implies a good solvency position, hence leading to high stability for the bank. We use the natural logarithm of the *Z-score* to control for outliers (Elnahass et al. 2021). Following Trinh et al. (2020a), we calculate the standard deviation of return on assets over the entire sample period.^6^

Furthermore, we examine bank financial performance using cost efficiency and two profitability measures. We measure the bank cost efficiency through the cost to income ratio (*COST/INCOME*), which reflects overhead costs relative to gross revenues. A higher ratio suggests lower levels of a bank’s operating efficiency (Abdelsalam et al. 2020). Moreover, we utilise two accounting-based profitability measures: return on average assets (*ROAA*) and return on average equity (*ROAE*). The higher the reported *ROAE* and *ROAA*, the better the profitability performance of a bank. These measures are more robust to assess the bank's financial performance by gauging the extent of operational efficiency and capturing the nuances of the bank’s diversifying earnings through non-interest income activities as well as the cost controls (Mollah and Zaman 2015; Elyasiani and Zhang 2015).

### Measures of the global terrorism index (GTI)

We measure terrorism risk utilising the Global Terrorism Index (GTI), which is a comprehensive measure analysing the impact of terrorism for 163 countries and covers 99.7% of the world’s population. The impact of terrorism is measured by a scale from 0 to 10, where 0 is no impact and 10 is a very high impact (Alam 2013; Arif and Suleman 2017). The index is produced by the Institute for Economics and Peace (IEP) based on data from the Global Terrorism Database (GTD). Data for the GTD is collected by the National Consortium for the Study of Terrorism and Responses to Terrorism (START) at the University of Maryland. The GTD contains over 170,000 terrorist incidents for the period 1970–2018.

### Empirical models

This study employs panel data analysis to account for the unobservable and constant heterogeneity, i.e., management style, business strategy, or other bank-specific features (Andres and Vallelado 2008). However, some independent variables in the model (e.g., board of directors and audit of the committee) are determined simultaneously with dependent variables, leading to possible simultaneity bias. Endogeneity is a common issue in banking and corporate governance studies (Abdallah and Ismail 2017). To mitigate potential endogeneity between terrorism risk and bank stability, we use the three-Stage Least-Square (3SLS) following prior studies (e.g., Elyasiani and Zhang 2015; Elnahass et al. 2020a; Trinh et al. 2020a).^7^ We also use instrumental variables following Ferris et al. (2003), Mollah and Zaman (2015) and Trinh et al. (2020a). Our applied methodology controls for three types of endogeneity: dynamic endogeneity, heteroscedasticity and autocorrelation.

We select three main Instrumental Variables (IVs) for terrorism risk. Our first IV is the control of the corruption country index (source: World Bank). This IV reflects the perceptions of petty/grand forms of corruption. Higher values infer better control of corruption. Shelley (2004) and Simpson (2014) report that terrorism risk is positively related to corruption in the same country, where Boussiga and Ghdamsi (2016) find a co-integration relationship between corruption and terrorism. In line with Trinh et al. (2020a), our second IV for terrorism risk is the country-level income-generating category (recorded in World Bank), which is a dummy variable taking a value of 1 if the ‘home’ bank is in a country classified as a middle- or high-income-generating nation, and 0 otherwise.^8^ Terrorism is concentrated in low-income countries with limited economic opportunities, a high rate of unemployment, and limited opportunities for people to upgrade their economic and social class (see Muller and Seligson 1987; De Mesquita 2005; Estrada et al. 2015). The empirical literature documents a positive association between economic depressions and terrorism (Blomberg et al. 2004; Drakos and Gofas 2004; De Mesquita 2005). Following the literature, we argue that a country with a developed economic system and a high level of income is likely to have a decrease in terrorist operations. Our final IV for terrorism risk is the rule of law that reflects perceptions of the extent to which agents have confidence in and abide by the rules of society and in particular the quality of contract enforcement, property rights, the police and the courts, as well as the likelihood of crime and violence. Its value ranges between − 2.5 (weak) and + 2.5 (strong) governance performance (World Bank 2016). Choi (2010) documents that a high-quality rule of law dampens the likelihood of any type of terrorist event. In the same vein, Neuman (2004) and Macdonald et al. (2019) emphasise the importance of rule of law in preventing and compacting terrorist attacks. We therefore expect that terrorism risk is negatively related to the rule of law in the same country.

The three IVs are correlated with the possible endogenous variables (i.e., GTI) and should predict bank performance/risk only indirectly through their effects on the endogenous variables.^9^ These IVs can indirectly affect bank performance/risk because the country-level indicators are less likely to influence an individual bank’s performance and risk-taking endogenously.

For our two study hypotheses (*H*_01_ and *H*_02_), we treat both terrorism risk and bank stability as endogenous variables and specify simultaneous equation models, Eqs. (1) and (2). The first equation, Eq. (1), estimates the impact of terrorism risk on bank financial risk, while the second equation estimates the impact of terrorism risk on bank financial performance as follows:

Model (1) examines the effect of Global Terrorism on bank financial risk:

1 $${{{Risk }_{it}={{\beta }_{0}}_{it}+{\beta }_{1}{ Terrorism\,risk }_{it}}+ \phi control +\mu Contry\,effects+ \alpha Year\,effects+}{\varepsilon }_{it}$$

Model (2) examines the effect of Global Terrorism on bank financial performance:

2 $${{{Performance }_{it}={{\beta }_{0}}_{it}+{\beta }_{1}Terrorism\,risk{ }_{it} }+ \phi Control+\mu Country\,effects+\alpha Year\,effects+}{\varepsilon }_{it}$$

where *Risk*_it_ represents {*LLR/GR, LA/DSF* and *Z-score*}. *Performance*_*it*_ represents {*COST/INCOME, ROAA* and *ROAE*}; Terrorism risk_it_ represents the Global Terrorism Index (*GTI*); ϕControl is a vector of control variables in the performance model. ε_it_ is the error term.

We control for a set of bank-level characteristics that are commonly related to financial performance. In order to account for country-specific group heterogeneity, it is necessary to employ country-fixed effects (Vita and Luo 2018; Fernández et al. 2016), because they are linked to the financial stability of the banks. Our control variables comprise: board size (*LogBSIZE*), to capture the board’s role and effectiveness (Faleye and Krishnan 2017; Arnaboldi et al. 2020; Marie et al. 2021); board independence (*%INDEP*) (Faleye and Krishnan 2017; Marie et al. 2021); board meeting (*BMEET*) (Liang et al. 2013; Dong et al. 2017); and CEO duality (*DUAL*) (Mollah et al. 2017; Faleye and Krishnan 2017). Moreover, we control for institutional ownership (*INSTITOWNER*) (Marie et al. 2021); and audit committee size (*ACSIZE*) (Choi et al. 2004). We also control for bank age (*LogAge*), which reflects the bank’s experience and informational advantages, measured by the difference between the sample year and the bank’s establishment year (Elnahass et al. 2021). We control for the bank size (*LogTA*) (Elnahass et al. 2020b). Following Arnaboldi et al. (2020) and Trinh et al. (2020a), we use the total equity-to-total asset ratio (*TE_TA*) as a measure of solvency and this is determined based on information derived from a business’s operations balance sheet. Furthermore, we use the ratio Net Loans/Total Assets (*NL/TA*) to control for the percentage of the bank’s assets, which are tied up in loans. The higher this ratio, the less liquid the bank will be. Moreover, we control for the ratio of Impaired Loans to Gross Loans (*IL_GL*), which measures the number of total loans that are doubtful. The lower the ratio, the higher the quality of the assets.

We include the inverse of log (*Z-score*) (*1/z*) in all the operating performance models to capture the positive effect of risk-taking on the bank’s performance (Mollah and Zaman 2015; Trinh et al. 2020a). We include the *COST/INCOME* in all the bank risk models to capture bank cost efficiency (Abedifar et al. 2013; Abdelsalam et al. 2020). Lower cost efficiency will increase bank risks because inefficiency illustrates a poorly run bank that has more risk-taking incentives. We control for the bank’s listing status using a dummy variable (*LISTED*) taking the value of 1 if the bank is listed and 0 if it is unlisted (Elnahass et al. 2018). Moreover, we use the annual growth rate in the gross domestic product (*GDP_GROWTH*) to identify the economic development of the region/country (e.g., Abedifar et al. 2013; Arnaboldi et al. 2020); Finally, we control for the country inflation rate (*INFLA*) (e.g., Vita and Luo 2018; Fernández et al. 2016). Table 2 presents variable definitions and notations in our models.

**Table 2:** Variable definitions

Variables	Abbreviations	Definitions
Credit Risk	LLR/GR	The ratio of loan loss reserves to gross loans. The higher the ratio, the higher the credit risk
Bank Liquidity Risk	LA/DSF	The ratio of liquidity assets to deposits and short-term funding
Insolvency Risk	Z-score	The distance to default, which is calculated as a sum of the Return on Assets (ROA) plus Capital Assets Ratio (CAR) scaled by the standard deviation of ROA. The higher the log of Z-score, the lower the insolvency risk
Return on Average Equity	ROAE	Net income divided by average total equity
Return on Average Asset	ROAA	The return on assets (ROA), which is net income divided by total assets
Cost Inefficiency	COST/INCOME	Cost to Income ratio
Global Terrorism Index	GTI	Global Terrorism Index within countries measured by scale 0 to 10; 0 is no impact but 10 is a very high impact
Board of Directors Size	BSIZE	Natural logarithm of the total number of board of directors’ members
Board Meeting	BMEET	Measured by the number of board of directors’ meetings within the year
Board Independence	%INDEP	Percentage of independent non-executive directors on the board of directors
CEO Duality	DUAL	Dummy variable, 1 if the CEO is also the Chairman of the board of directors; otherwise 0
Institutional Ownership	INSTITOWNER	The total number of shares owned by institutions divided by the total number of shares owned by banks
Audit Committee Size	ACSIZE	The audit committee size for each bank year is calculated based on the number of the audit committee members
Bank Size	LogTA	Natural logarithm of total assets of a bank at the end of the year
Equity to Total Assets	TE_TA	The ratio of Equity to Total Assets. The higher the TE_TA, the lower the level of a bank’s capital risk
Impaired Loans/Gross Loans	IL_GL	The amount of total loans that are doubtful. The lower this figure is, the better the quality of the asset
Net Loans/Total Assets	NL_TA	The percentage of the assets of the bank tied up in loans. The higher this ratio, the less liquid the bank will be
Bank Risk-Taking	1/Z	Bank risk-taking behaviour is calculated by the inverse of LogZscore
Listed Bank	LISTED	Dummy variable: 1 if the bank is listed on a stock market, 0 otherwise
Bank Age	LogAge	Natural logarithm of the difference between the sample year and the year of a bank’s first appearance
Political Stability	PStability	Political Stability and Absence of Violence. Its value ranges between -2.5 (weak) and + 2.5 (strong) governance performance
GDP Growth Rate	GDP	Annual Gross Domestic Product (GDP) growth rate
Inflation Rate	INFLA	Inflation rate within countries

This table presents definitions and measurements of all variables employed in models tested

### Descriptive statistics

Table 3 presents descriptive statistics for the entire sample and two sub-samples for banks located in high terrorism risk countries (HTRc) and low terrorism risk countries (LTRc).^10^ The full sample shows comparable statistics to prior studies (e.g., Mollah and Zaman 2015; Trinh et al. 2020a) concerning bank risk and financial performance indicators. The average mean (median) of the Global Terrorism Index (GTI) is 3.16 (4.21). Regarding the two sub-samples, banks in HTRc report riskier bank profiles than in LTRc, with a higher mean of LLR/GR (i.e., higher credit risk), and a higher mean of *LA_DF* (i.e., lower liquidity risk) and a lower mean of *Z-score* (i.e., higher insolvency risk). Banks facing economic and financial stress encounter high credit risk and liquidity management issues (Čihák and Hesse 2010). The results showing that HTRc banks have a lower liquidity risk can be justified by the huge bailouts injected by the governments in countries where terrorism events are frequent occurrences to breathe life into the economy. Also, this is consistent with the ‘normalisation of terror’ hypothesis, as having a high number of terrorism incidents becomes a routine element over time, leading to the erosion of sensitivity to prolonged terrorism (see Arin et al. 2008; Peleg et al. 2011; Shahbaz 2013; Alam 2013).

**Table 3:** Descriptive statistics

Full sample									High Terrorism Sample Mean	Low Terrorism Sample Mean	Two-Sample t-Test (two-tailed)
Variables/ratios	N	Mean	Median	Std	Min	0.25	0.75	Max			
LLR/GR	954	1.46	1.45	0.88	− 0.32	0.88	1.95	3.77	1.57	1.33	− 4.27 ***
LA_DF		3.45	3.41	0.62	1.89	3.01	3.85	5.60	3.59	3.26	− 8.63 ***
Z-score		4.03	4.09	1.33	1.03	3.05	4.99	7.07	3.87	4.23	4.11 ***
COST/INCOME		3.75	3.72	0.40	2.55	3.50	4.03	4.82	3.83	3.65	− 7.17 ***
ROAA		1.44	1.42	1.20	− 2.67	0.81	1.94	6.54	1.33	1.59	3.37 ***
ROAE		2.36	2.45	0.74	− 1.77	2.10	2.76	3.88	2.40	2.32	− 1.67 *
GTI		3.61	4.21	2.64	0.00	0.58	5.66	8.62	5.65	0.948	− 58.14 ***
BSIZE		9	9	0.10	5	8	11	15	9	9	− 1.26
BMEET		7	6	0.15	4	5	7	16	7	7	− 1.08
%INDEP		0.63	0.63	0.21	0.15	0.45	0.80	1.00	0.63	0.61	− 1.45
DUAL		0.11	0.00	0.32	0.00	0.00	0.00	1.00	0.17	0.04	− 6.01 ***
INSTITOWNER		0.79	0.86	0.20	0.31	0.64	0.97	1.00	0.81	0.77	− 3.27 **
ACSIZE		4	3	0.09	3	3	4	6	4	4	2.08 **
LogTA		5.48	5.47	0.28	5.01	5.23	5.71	6.00	5.47	5.48	0.51
TE_TA		2.51	2.50	0.47	1.58	2.23	2.72	4.32	2.40	2.66	9.06 ***
IL_GL		1.50	1.42	1.07	− 0.86	0.78	2.18	4.07	1.56	1.42	− 1.97 *
NL_TA		3.79	3.98	0.59	1.17	3.57	4.18	4.38	3.62	4.01	10.93 ***
LISTED		0.65	1.00	0.48	0.00	0.00	1.00	1.00	0.53	0.81	9.36 ***
LogAge		1.61	1.62	0.22	0.95	1.56	1.76	2.08	1.66	1.56	− 6.70 ***
PStability		− 0.68	− 0.87	1.01	− 3.00	− 1.44	0.17	1.22	− 1.36	0.22	38.53 ***
GDP		0.03	0.03	0.02	− 0.00	0.02	0.04	0.08	0.03	0.04	3.67 ***
INFLA		0.03	0.03	0.08	− 0.13	− 0.02	0.09	0.21	0.04	0.02	− 4.62 ***

The table presents descriptive statistics of all variables used in the regression models of the study. The sample period is between 2010 and 2018. The Std. is the standard deviation. Min and Max are the minimum and maximum values of each variable, respectively. The N is the number of bank-year observations. We also report on the paired sample mean test (T-test). The ***^,^**^,^* represent *p*-values of 0.01, 0.05, and 0.10. See Table 2 for variable definitions

Moreover, banks in HTRc also report higher average profitability relative to banks in LTRc, with higher means for both ROAE and ROAA. Banks in HTRc have a higher average cost (lower operating efficiency) than those in LTRc, with higher means for *COST/INCOME* ratio. The two-sample t-test shows that banks within HTRc on average hold a better financial performance but they engage in more risk-taking than those in LTRc. Moreover, the two-sample t-test shows a significant difference between these two sub-samples for the GTI. This finding is consistent with the trade-off between risk and return theory, as banks require higher returns when they bear higher risk (see Hughes et al. 2001; Mester 2008). For the corporate governance controls, banks in HTRc report a higher mean of BOD size (*BSIZE*) relative to LTRc, while we cannot find a significant difference in the board meeting numbers (*BMEET*) or board independence (*%INDEP*) between the two sub-samples. Furthermore, for institutional ownership (*INSTITOWNER*), banks in HTRc (LTRc) report 81% (77%) respectively, with a significant difference between the two sub-samples. Finally, banks located in HTRc show a higher mean of audit committee size (*ACSIZE*) relative to those in LTRc. Our sampled banks located in both HTRc and LTRc report relatively similar bank size (*LogTA*); banks in LTRc are significantly younger in age (*LogAge*) and appear to be less liquid (i.e., higher *NL_TA* ratio) than banks located in HTRc. The Pearson correlation coefficients of all dependent and independent variables affirm that multicollinearity does not appear to be a serious statistical problem.^11^

## Empirical results

### Terrorism risk and bank stability

In Table 4, we report the three-stage least squares (3SLS) estimations for the full sample. Panel A shows the results for bank risk, while Panel B presents the results for financial performance. In Panel A, we find that the GTI is positively associated with credit risk (*LLR_GR*) and liquidity ratio (*LA/DSF*). Moreover, GTI is negatively associated with the insolvency ratio (*Z-score*). These results indicate high insolvency risk and credit risk, but low liquidity risk for banks operating under high terrorism risk exposure. In terms of economic significance, the coefficients on the different risk indicators are also economically significant. 1% increase in GTI, leads to 12.1% increase in the ratio of loan loss reserve to gross loan while reduce the bank distance to default by 0.261. These findings suggest that banks with high terrorism risk exposure exhibit an overall high-risk profile, and hence have low financial stability. However, 1% increase in GTI will enhance the bank ratio of liquidity assets to deposits and short-term funding by 10.7%. These findings can be explained through the economic consequences of terrorism that adversely affect economic stability (e.g., economic growth, foreign direct investment (FDI), domestic investments, consumption plans and government spending), which overall seem to have detrimental impacts on bank stability (Boateng et al. 2015; Ozili 2018). Financial stability is highly impacted by the psychological terror caused by terrorism events, which have a negative influence on investor confidence and investment decisions. Terrorist attacks increase the volatility of financial markets, which leads to high risk-taking and low financial stability for banks (see Estrada et al. 2015; Younas 2015; Arif and Suleman 2017; El Ouadghiri and Peillex 2018). Our findings are also in line with the political economy theories (Caporaso and Levine 1992) which suggest that political instability affects different macroeconomic factors and any political shocks will be transmitted to the economy, affecting the economic activities and risk exposure. Another explanation for the different associations between terrorism and different measures of risk is the technical nature of each risk measure used. The economic downturn due to terrorism and its severe impact on business cash flows will drive the bank to increase the loan loss reserves and to follow a restricting policy in giving loans. This will increase *LLR/GR,* indicating a higher credit risk for a bank, leading to high insolvency risk. Moreover, banks under a lack of investment and consumer and government spending will be left with high liquid assets by investing in short-term governmental securities. Furthermore, the negative association between terrorism index and liquidity risk can be explained by the substantial bailouts injected by a government to help the economy to overcome the terrifying economic and financial consequences of terrorist attacks. Also, our results are consistent with the ‘normalisation of terror’ hypothesis, as having a high number of terrorism incidents becomes a routine element over time, leading to the erosion of depositors’ sensitivity to prolonged terrorism (see Peleg et al. 2011; Shahbaz 2013; Alam 2013). The overall findings support our first hypothesis, ***H***_***01***_, indicating that terrorist attacks significantly increase the risk exposure of banks in the MENA region.

**Table 4:** The effect of terrorism risk on bank stability

Variables	Panel A			Panel B		
	Financial risk			Financial performance		
	(1)	(2)	(3)	(4)	(5)	(6)
	LLR_GR	LA_DF	Z-score	Cost_Income	ROAA	ROAE
GTI	0.121**	0.107***	− 0.261***	− 0.198*	− 0.0121	0.156**
	(0.044)	(0.037)	(0.086)	(0.087)	(0.090)	(0.056)
BSize	0.226	− 0.109	− 0.147	− 0.363**	1.547***	0.973***
	(0.209)	(0.178)	(0.449)	(0.148)	(0.468)	(0.262)
BMEET	0.244	− 0.306*	0.043	0.292**	0.427	− 0.034
	(0.158)	(0.134)	(0.339)	(0.106)	(0.354)	(0.196)
%INDEP	− 0.052	− 0.103	0.126	0.249***	− 0.157	0.029
	(0.092)	(0.078)	(0.197)	(0.061)	(0.205)	(0.114)
DUAL	− 0.087	− 0.147***	0.280**	− 0.026	0.041	0.080
	(0.058)	(0.050)	(0.126)	(0.039)	(0.132)	(0.073)
INSTITOWNER	− 0.055	− 0.066	0.314	0.056	0.027	0.239*
	(0.095)	(0.081)	(0.204)	(0.065)	(0.214)	(0.119)
ACSIZE	− 0.161	− 0.026	0.145	− 0.479***	0.486	0.353
	(0.197)	(0.168)	(0.424)	(0.131)	(0.441)	(0.245)
LOGTA	− 0.027	0.018	− 0.328**	0.056	0.304*	0.106
	(0.056)	(0.047)	(0.120)	(0.040)	(0.125)	(0.070)
TE_TA	0.150**	0.304***	− 0.207*	− 0.042	0.844***	− 0.150**
	(0.046)	(0.039)	(0.098)	(0.032)	(0.103)	(0.057)
IL_GL	0.398***	0.030*	− 0.048	0.006	− 0.238***	− 0.077***
	(0.019)	(0.016)	(0.041)	(0.014)	(0.043)	(0.024)
NL_TA	− 0.259***	− 0.323***	0.282***	− 0.056*	− 0.319***	0.036
	(0.037)	(0.032)	(0.079)	(0.025)	(0.083)	(0.046)
Cost_Income	− 0.055	− 0.108**	0.077	–	–	–
	(0.048)	(0.040)	(0.102)	–	–	–
1/Z	–	–	–	− 0.049	0.203	0.047
	–	–	–	(0.065)	(1.028)	(0.122)
LISTED	− 0.055	− 0.089**	0.276***	0.0127	− 0.184**	− 0.135**
	(0.040)	(0.034)	(0.086)	(0.027)	(0.090)	(0.051)
LogAge	0.131	0.115	0.212	0.061	0.018	0.336***
	(0.090)	(0.076)	(0.193)	(0.060)	(0.202)	(0.112)
PStability	− 0.630***	0.072	0.251	− 0.022	− 0.482**	− 0.356***
	(0.086)	(0.072)	(0.181)	(0.089)	(0.189)	(0.107)
GDP	− 2.611*	0.206	0.682	− 1.015	13.59***	5.041***
	(1.181)	(1.001)	(2.500)	(1.146)	(2.614)	(1.477)
INFLA	− 1.154***	0.108	− 0.243	0.377	2.363***	0.797*
	(0.326)	(0.276)	(0.692)	(0.302)	(0.726)	(0.409)
Constant	0.896	4.457***	5.001***	4.415***	− 4.136***	− 0.920
	(0.509)	(0.433)	(1.094)	(0.326)	(1.047)	(0.588)
Observations	954	954	954	954	954	954
Year effects	Yes	Yes	Yes	Yes	Yes	Yes
Country effects	Yes	Yes	Yes	Yes	Yes	Yes
R^2^	0.712	0.489	0.239	0.206	0.269	0.161
Wald Chi2	435 ***	327 ***	217 ***	397 ***	345 ***	364 ***
LM Statistics (*p*-value)	000	000	000	000	000	000
Sargan test (*p*-value)	536	463	745	413	374	295

The table presents Three-Stage Least-Squares (3SLS) estimations for the full sample of banks identifying the impact of terrorism risk on a bank’s financial stability, which is represented by bank risk as measured through the credit risk, liquidity risk and insolvency risk {Z-score, LLR/GR and LA/DSF} in Panel A, and bank performance as measured through profitability (ROAA and ROEA), COST/INCOME ratio (Panel B). Terrorism risk represents the Global Terrorism Index (GTI P-values in parentheses, *p < 0.10, **p < 0.05, ***p < 0.01. LM) and Sargan test shows that our models are correctly identified, and our selected IVs are valid

Results from examining bank financial performance indicators, in Panel B, indicate that the coefficients of GTI are negatively related to *COST/INCOME* but positively associated with *ROAE*. Our results are also economically significant, 1% increase in GTI will decrease the cost to income ratio by 19.8% and increase ROAE by 15.6%. These results indicate that high exposure to terrorist attacks is associated with a high profitability position and better cost efficiency for banks. The results also confirm predictions for the improved financial performance of banks during the period of attacks under our second hypothesis, ***H***_***02.***_ Such increases in profitability can be explained by the impact of economic stimulation package, interest rate cuts and liquidity injections after the terrorist attacks, which may support the recovery of the financial system. Our results are consistent with Arif and Suleman (2017), who report a positive relationship between terrorist index and returns for financial institutions. Another explanation for the positive association between the terrorist attacks and bank profitability can be justified through the modern portfolio theory, which assumes a trade-off between risk and return. Terrorist attack as a part of the political risk is a systematic risk that should be priced (Belkhir et al. 2019). The increase of the risk exposure of banks because of terrorism will drive banks to require a higher profit margin as a compensation for the risk [for discussion about the risk-return trade-off in banks see Demirgüç-Kunt (1989) and Anginer et al. (2014)]. Post a terrorist attack, usually, the rescue packages tighten the interest rates offered by banks, which seems to reduce banks’ costs. However, banks tend to increase the interest rate margin to compensate for the increase in the credit risk of their customers. On the other hand, Gaibulloev and Sandler (2008) and Estrada et al. (2015) find that counter-terrorism strategies might increase aid flow and other interventions into a country, which in turn increase the demand on bank services.

### Countries with high versus low terrorism risk exposure

Based on our findings above, the impact of terrorism risk on bank stability could differ among countries that are characterised by high (low) exposure to risk of terrorism. Therefore, in Table 5 we extend our examination to two sub-samples representing banks located within high terrorism risk countries (HTRc) in Panel A and those operating in low terrorism risk countries (LTRc) in Panel B.

**Table 5:** The effect of terrorism risk on bank stability: high and low terrorism risk countries

Variables	Panel A						Panel B					
	High Terrorism Risk Countries (HTRc)						Low Terrorism Risk Countries (LTRc)					
	Financial Risk			Financial Performance			Financial Risk			Financial Performance		
	(1)	(2)	(3)	(4)	(5)	(6)	(7)	(8)	(9)	(10)	(11)	(12)
	LLR_GR	LA_DF	Z–score	Cost_Income	ROAA	ROAE	LLR_GR	LA_DF	Z-score	Cost_Income	ROAA	ROAE
GTI	− 0.273*	0.124*	− 0.513***	− 0.287***	0.548***	0.407***	0.356***	0.097	0.046	0.127**	− 0.519***	− 0.254 *
	(0.127)	(0.072)	(0.178)	(0.082)	(0.142)	(0.086)	(0.097)	(0.108)	(0.209)	(0.059)	(0.150)	(0.137)
BSize	− 0.207	− 0.132	0.126	− 0.599**	2.432***	1.087***	0.323	− 0.575	− 1.112 *	− 0.254	− 1.536**	0.225
	(0.285)	(0.221)	(0.588)	(0.187)	(0.602)	(0.287)	(0.320)	(0.362)	(0.648)	(0.190)	(0.553)	(0.465)
BMEET	0.455 *	− 0.643***	0.218	− 0.047	0.522	0.372	− 0.310*	− 0.170	0.414	0.494***	0.512	− 0.363
	(0.260)	(0.201)	(0.534)	(0.171)	(0.547)	(0.255)	(0.183)	(0.207)	(0.362)	(0.105)	(0.313)	(0.259)
%INDEP	− 0.035	0.088	0.447	0.376***	− 0.252	0.179	0.084	− 0.223 *	− 0.328	0.164**	− 0.152	− 0.098
	(0.147)	(0.114)	(0.304)	(0.096)	(0.307)	(0.143)	(0.109)	(0.124)	(0.217)	(0.064)	(0.191)	(0.157)
DUAL	− 0.133*	− 0.160***	0.307**	− 0.071	0.035	0.071	0.181 *	− 0.008	0.140	0.088	0.195	0.133
	(0.070)	(0.054)	(0.144)	(0.046)	(0.147)	(0.068)	(0.101)	(0.115)	(0.200)	(0.060)	(0.178)	(0.147)
INSTITOWNER	0.081	− 0.010	0.538*	0.101	0.050	0.252 *	− 0.228 *	− 0.009	0.219	− 0.100	0.505**	0.617***
	(0.143)	(0.111)	(0.295)	(0.094)	(0.302)	(0.141)	(0.126)	(0.143)	(0.249)	(0.074)	(0.222)	(0.183)
ACSIZE	− 0.642**	− 0.717***	0.346	− 0.628***	1.804***	0.396	0.418	0.617 ***	− 0.415	− 0.298 *	− 0.853 *	0.137
	(0.275)	(0.214)	(0.567)	(0.180)	(0.577)	(0.267)	(0.269)	(0.305)	(0.532)	(0.158)	(0.473)	(0.390)
LogTA	0.062	0.072	− 0.224	0.063	0.119	− 0.023	− 0.139*	0.029	− 0.459***	0.067	0.667***	0.287**
	(0.077)	(0.060)	(0.159)	(0.051)	(0.163)	(0.076)	(0.071)	(0.080)	(0.146)	(0.042)	(0.125)	(0.103)
TE_TA	0.188***	0.381***	− 0.351**	− 0.066	0.870***	− 0.074	− 0.020	0.260***	− 0.011	− 0.088**	0.555***	0.288***
	(0.064)	(0.050)	(0.133)	(0.042)	(0.139)	(0.063)	(0.060)	(0.068)	(0.118)	(0.035)	(0.105)	(0.086)
IL_GL	0.375***	− 0.008	− 0.058	0.001	− 0.164***	− 0.015	0.415***	0.094**	0.065	0.053***	− 0.291***	− 0.253***
	(0.024)	(0.019)	(0.050)	(0.016)	(0.051)	(0.024)	(0.032)	(0.036)	(0.063)	(0.019)	(0.054)	(0.045)
NL_TA	− 0.359***	− 0.381***	0.369***	− 0.030	− 0.421***	0.020	− 0.053	− 0.285***	0.236**	− 0.123***	0.125	0.138 *
	(0.048)	(0.038)	(0.10)	(0.032)	(0.102)	(0.048)	(0.055)	(0.062)	(0.110)	(0.032)	(0.095)	(0.078)
Cost_Income	− 0.205***	− 0.075	− 0.080	–	–	–	0.157*	− 0.274***	0.247	–	–	–
	(0.061)	(0.046)	(0.122)	–	–	–	(0.082)	(0.093)	(0.164)	–	–	–
1/Z	–	–	–	0.020	− 0.537	− 0.039	–	–	–	− 0.095	− 0.252	− 0.284
	–	–	–	(0.0729)	(0.274)	(0.108)	–	–	–	(0.116)	(0.524)	(0.184)
LISTED	− 0.103*	− 0.131***	0.348***	0.071*	− 0.069	− 0.118*	0.067	− 0.079	0.292**	− 0.100**	− 0.327***	− 0.096
	(0.052)	(0.040)	(0.108)	(0.035)	(0.109)	(0.052)	(0.063)	(0.071)	(0.127)	(0.037)	(0.110)	(0.091)
LogAge	− 0.028	− 0.014	0.169	0.153 *	− 0.291	0.138	0.422***	0.443***	0.267	− 0.022	0.849***	0.669***
	(0.129)	(0.101)	(0.267)	(0.085)	(0.273)	(0.127)	(0.111)	(0.126)	(0.220)	(0.065)	(0.196)	(0.161)
PStability	− 0.939***	0.067	0.354	− 0.079	− 0.490*	− 0.352***	− 0.663***	− 0.231	0.185	− 0.067	0.338	− 0.019
	(0.143)	(0.095)	(0.246)	(0.094)	(0.235)	(0.121)	(0.165)	(0.186)	(0.332)	(0.097)	(0.283)	(0.238)
GDP	− 8.505***	− 0.345	− 3.352	− 3.696**	20.35***	6.661***	− 0.544	1.107	− 1.051	− 1.485	5.042 *	3.200
	(1.988)	(1.515)	(4.007)	(1.316)	(4.099)	(1.968)	(1.628)	(1.842)	(3.285)	(0.962)	(2.826)	(2.353)
INFLA	− 0.213	− 0.252	− 1.074	1.528***	0.787	1.086 *	− 1.602**	0.295	0.340	− 0.10	1.316	− 0.091
	(0.675)	(0.524)	(1.394)	(0.443)	(1.412)	(0.657)	(0.567)	(0.637)	(1.188)	(0.339)	(0.946)	(0.816)
Constant	0.368***	0.910***	0.283***	0.350***	0.979	0.082***	0.127	0.984***	0.587*	− 1.273	0.915***	− 0.437
	(0.893)	(0.686)	(2.154)	(0.457)	(1.637)	(1.148)	(0.690)	(0.591)	(1.477)	(0.792)	(0.465)	(2.280)
Observations	539	539	539	539	539	539	415	415	415	415	415	415
Year Effects	Yes	Yes	Yes	Yes	Yes	Yes	Yes	Yes	Yes	Yes	Yes	Yes
Country Effects	Yes	Yes	Yes	Yes	Yes	Yes	Yes	Yes	Yes	Yes	Yes	Yes
R^2^	0.785	0.650	0.436	0.239	0.256	0.208	0.510	0.389	0.103	0.438	0.171	0.205
Wald Chi2	253***	536***	492***	384***	364***	512***	427***	254***	557***	211***	432***	425***
LM Statistics (*p*-value)	000	000	000	000	000	000	000	000	000	000	000	000
Sargan test (*p*-value)	453	724	653	284	352	298	539	553	634	487	628	294

This table presents the results for different levels of Terrorism risk countries which are classified as High Terrorism risk countries (HTRc) (Panel A) and Low Terrorism risk countries (LTRc) (Panel B) across conventional banks in the MENA region. The table presents Three-Stage Least-Squares (3SLS) estimations for the full sample of banks identifying the impact of terrorism risk on a bank’s financial stability, which is represented by bank risk as measured through the credit risk, liquidity risk and insolvency risk (Panel A), and bank performance as measured through profitability (ROAA and ROEA), COST/INCOME ratio (Panel B). *P*-values in parentheses, *p < 0.10, **p < 0.05, ***p < 0.01. LM and Sargan tests show that our models are correctly identified, and our selected IVs are valid. See Table 2 for variable definitions

The results in Panel A confirm our main finding suggesting a positive association between insolvency risk and risk of terrorism for countries with high risk exposure. In addition, we find that a high risk of terrorist attacks significantly increases financial performance for banks located in these countries. This is due to a negative coefficient on the *COST/INCOME* ratio and a positive coefficient on both ROAE and ROAA ratios, which are also economically significant. In contrast, the results in Panel B (i.e., banks in countries with low risk exposure to terrorism) report a significantly high credit risk but these banks have low financial performance, in terms of cost efficiency and profitability*.* Findings remain to be economically significant in line with the main findings.

Taken together, these findings indicate that the differential impacts of terrorism on bank stability are conditional on the degree of exposure to terrorism risk across MENA countries. Banks located in HTRc tend to require a higher return on their loans and investment as compensation for increased insolvency risk, while those operating in LTRc tend to have some difficulties in generating enough income, acquiring governmental financial aid and/or utilising their financial assets and capital to absorb this political shock. Hence, banks located in countries with low exposure to terrorism exhibit poorer cost efficiency and profitability when compared to those within countries with high exposure. These results are in line with our predication and are supported by the ‘normalisation of terror’ hypothesis (Arin et al. 2008; Peleg et al. 2011).

## Additional analyses and robustness checks

### The impact of terrorism within high (low) income-generating countries

According to the World Bank, the world's economies can be classified into four income groups^12^: high, upper-middle, lower middle and low. Therefore, we follow this classification for our full sample in order to assess whether the country income level mediates the impact of terrorism on bank stability. Panel A in Table 6 reports the results for high-income-generating countries (comprising high and upper-middle), and Panel B shows the results of low-income-generating countries (comprising low and lower middle).

**Table 6:** Terrorism risk and bank stability: high- versus low-income generating countries

Variables	Panel A						Panel B					
	High income						Low income					
	Financial risk			Financial performance			Financial risk			Financial performance		
	(1)	(2)	(3)	(4)	(5)	(6)	(7)	(8)	(9)	(10)	(11)	(12)
	LLR_GR	LA_DF	Z-score	Cost_Income	ROAA	ROAE	LLR_GR	LA_DF	Z-score	Cost_Income	ROAA	ROAE
GTI	0.178***	0.047	0.121 *	0.026	− 0.179***	− 0.073 *	− 0.683**	0.441***	− 0.511	− 0.316**	0.840*	0.614***
	(0.034)	(0.038)	(0.067)	(0.019)	(0.058)	(0.043)	(0.263)	(0.135)	(0.478)	(0.119)	(0.443)	(0.208)
BSize	0.072	− 0.763*	− 0.249	− 0.370**	− 0.006	1.038**	0.0002	− 0.042	− 0.673	− 0.349*	1.827**	0.651*
	(0.276)	(0.316)	(0.544)	(0.164)	(0.493)	(0.373)	(0.366)	(0.218)	(0.638)	(0.188)	(0.702)	(0.333)
BMEET	− 0.161	− 0.664***	− 0.055	0.178**	0.118	− 0.0344	1.056**	− 0.078	0.953	0.434	0.761	− 0.012
	(0.151)	(0.174)	(0.299)	(0.090)	(0.272)	(0.204)	(0.521)	(0.308)	(0.912)	(0.264)	(0.990)	(0.463)
%INDEP	0.014	0.122	0.039	0.218***	− 0.061	0.164	0.045	− 0.436***	0.702*	0.421***	− 0.481	− 0.045
	(0.102)	(0.117)	(0.201)	(0.060)	(0.181)	(0.136)	(0.231)	(0.138)	(0.403) **	(0.117)	(0.431)	(0.202)
DUAL	0.094	− 0.070	− 0.019	0.124	− 0.219	− 0.010	− 0.052	− 0.089*	0.331**	− 0.052	− 0.002	0.065
	(0.142)	(0.163)	(0.281)	(0.085)	(0.256)	(0.192)	(0.077)	(0.046)	(0.135)	(0.040)	(0.149)	(0.070)
INSTITOWNER	− 0.030	0.044	0.160	− 0.012	0.379*	0.397**	− 0.116	− 0.283**	0.275	0.116	− 0.191	0.069
	(0.105)	(0.120)	(0.208)	(0.062)	(0.189)	(0.142)	(0.207)	(0.124)	(0.362)	(0.106)	(0.397)	(0.186)
ACSIZE	0.477**	0.671**	0.709	− 0.173	− 0.530	0.092	− 1.040**	0.122	− 1.429*	− 0.761***	2.860***	0.410
	(0.228)	(0.261)	(0.458)	(0.136)	(0.410)	(0.308)	(0.377)	(0.224)	(0.659)	(0.191)	(0.708)	(0.330)
LogTA	− 0.097	− 0.093	− 0.334**	0.027	0.529***	0.234**	0.012	0.177***	− 0.213	0.032	0.084	− 0.063
	(0.064)	(0.073)	(0.132)	(0.038)	(0.116)	(0.087)	(0.099)	(0.060)	(0.173)	(0.051)	(0.191)	(0.090)
TE_TA	− 0.008	0.226***	− 0.204*	− 0.104***	0.314***	− 0.079	0.313***	0.327***	− 0.427**	− 0.083*	1.224***	− 0.123
	(0.053)	(0.060)	(0.106)	(0.031)	(0.094)	(0.070)	(0.089)	(0.055)	(0.156)	(0.046)	(0.177)	(0.082)
IL_GL	0.414***	0.071**	0.040	0.040**	− 0.234***	− 0.175***	0.359***	0.003	− 0.104**	− 0.016	− 0.204***	− 0.018
	(0.026)	(0.029)	(0.050)	(0.015)	(0.046)	(0.035)	(0.030)	(0.018)	(0.052)	(0.015)	(0.058)	(0.027)
NL_TA	− 0.291***	0.427***	0.082	− 0.132***	− 0.426***	0.040	− 0.256***	− 0.316***	0.551***	0.085**	− 0.291**	0.031
	(0.047)	(0.053)	(0.093)	(0.027)	(0.083)	(0.061)	(0.071)	(0.044)	(0.124)	(0.037)	(0.136)	(0.064)
Cost_Income	0.013	− 0.354***	0.067	–	–	–	− 0.286***	0.095*	− 0.215	–	–	–
	(0.063)	(0.073)	(0.126)	–	–	–	(0.083)	(0.048)	(0.145)	–	–	–
1/Z	–	–	–	− 0.129	0.253	0.253	–	–	–	0.095	0.238	− 0.120
	–	–	–	(0.105)	(0.864)	(0.238)	–	–	–	(0.067)	(0.136)	(0.118)
LISTED	− 0.029	− 0.204***	0.088	− 0.029	− 0.208**	− 0.091	− 0.104	0.006	0.483***	0.049	− 0.092	− 0.118 *
	(0.047)	(0.054)	(0.094)	(0.028)	(0.085)	(0.064)	(0.070)	(0.042)	(0.122)	(0.036)	(0.134)	(0.065)
LogAge	0.134	0.348**	− 0.015	− 0.106	0.576***	0.380**	0.344**	− 0.065	0.555**	0.274***	− 0.463	0.130
	(0.111)	(0.127)	(0.219)	(0.066)	(0.201)	(0.150)	(0.155)	(0.092)	(0.271)	(0.079)	(0.293)	(0.138)
PStability	− 0.376***	0.070	0.304	− 0.034	0.196	− 0.046	− 1.046***	0.125	0.697**	0.051	− 0.748**	− 0.399***
	(0.119)	(0.136)	(0.234)	(0.071)	(0.213)	(0.160)	(0.145)	(0.083)	(0.254)	(0.073)	(0.273)	(0.132)
GDP	0.034	− 0.004	2.025	− 0.496	2.130	1.339	− 5.220**	0.915	-10.10**	− 2.470**	23.94***	6.554***
	(1.298)	(1.487)	(2.597)	(0.768)	(2.327)	(1.747)	(2.315)	(1.372)	(4.056)	(1.189)	(4.527)	(2.174)
INFLA	− 1.326***	0.116	− 0.766	− 0.073	0.819	0.158	0.146	− 0.721	− 0.994	1.531***	1.168	0.997
	(0.377)	(0.431)	(0.743)	(0.225)	(0.678)	(0.511)	(0.863)	(0.513)	(1.498)	(0.441)	(1.623)	(0.762)
Constant	0.630**	0.616***	0.49***	2.274***	− 0.738	1.281	0.294	0.182**	0.279	0.582***	− 0.379***	− 2.173
	(0.921)	(0.483)	(2.341)	(0.637)	(2.110)	(1.236)	(1.263)	(0.497)	(2.641)	(0.735)	(2.264)	(1.635)
Observations	513	513	513	513	513	513	441	441	441	441	441	441
Year effects	Yes	Yes	Yes	Yes	Yes	Yes	Yes	Yes	Yes	Yes	Yes	Yes
Country Effects	Yes	Yes	Yes	Yes	Yes	Yes	Yes	Yes	Yes	Yes	Yes	Yes
R^2^	0.714	0.564	0.065	0.534	0.283	0.224	0.613	0.404	0.396	0.144	0.259	0.131
Wald Chi2	344 ***	510***	463***	274***	535***	482***	380***	274***	214***	284***	293***	264***
LM Statistics (*p*-value)	000	000	000	000	000	000	000	000	000	000	000	000
Sargan test (*p*-value)	510	483	763	634	294	646	506	483	435	651	730	573

This table presents the results for different rates of income of countries that are classified as high-income-generating countries (Panel A) and low-income-generating countries (Panel B) across conventional banks in the MENA region. The table presents Three-Stage Least-Squares (3SLS) estimations for the full sample of banks identifying the impact of terrorism risk on a bank’s financial stability, which is represented by bank risk as measured through the credit risk, liquidity risk and insolvency risk (Panel A), and bank performance as measured through profitability (ROAA and ROEA), COST/INCOME ratio (Panel B). *P*-values in parentheses, *p < 0.10, **p < 0.05, ***p < 0.01. LM and Sargan tests show that our models are correctly identified, and our selected IVs are valid. See Table 2 for variable definitions

The results in Panel A indicate that, for banks located in high-income-generating countries, high risk of terrorism is associated with low financial performance (i.e., significantly low *ROAA*) and high credit risk. In contrast, in Panel B, when banks in low income-generating countries are exposed to terrorist attacks they seem to exhibit low credit risk and low liquidity risk. Furthermore, this set of banks also report significantly high financial performance in terms of cost efficiency and profitability. These findings can be justified by the flight to safety and the flight to liquidity theory. In bad times, banks will prefer to invest in short-term securities and other liquid assets that will decrease the liquidity risk ratio (*LA/DF*). Moreover, the reported negative association between credit risk and terrorism is in line with Ashraf (2017) and Ozili (2020).

These findings suggest that high exposure to terrorist attacks in poor countries stifles credit market competition and dampens moral hazard problems, which promotes low risk-taking by banks. The variations in results among high- and low-income-generating countries are also in line with prior literature which documents that terrorism is more prolonged in poor economies (De Mesquita 2005; Estrada et al. 2015). Our results are also consistent with Essaddam and Karagianis (2014), who report that firms operating in wealthier or more democratic countries face greater volatility in stock returns relative to firms operating in developing countries due to terrorist attacks.

### Conditional value-at-risk (CoVaR): systematic risk

In this section, we examine the impact of terrorism attacks on an additional measure of financial stability: banks’ Conditional Value-at-Risk (CoVaR). This measurement of systemic risk is fundamental for the regulatory authorities monitoring and intervening in the financial system to ensure its stability (de Mendonça and da Silva 2018). Systemic risk is the risk of a collapse of the entire financial system, typically triggered by the default of one or more interconnected financial institutions (Borri et al. 2014). The CoVaR identifies the tail dependence between two different VaR (Value-at-Risk) distributions (De Mendonça and Da Silva 2018). Measuring the risk of the financial system through CoVaR can work as a check tool for the robustness of our results for measuring the impact of terrorism on the systemic risk and financial stability (Roengpitya and Rungcharoenkitkul 2011; Borri et al. 2014; Adrian 2016; Stolbov 2017; De Mendonça and Da Silva 2018; Hanif et al. 2019; Huang et al. 2019). CoVaR allows the quantification of systemic risk as to the impact of a financial institution, market or system on the value-at-risk (VaR) of other financial institutions, stock markets or systems (Huang et al. 2019). To construct annual CoVaR for each bank we use daily return prices of 72 listed commercial banks in the MENA region from July 24, 2010 to July 22, 2018. CoVaR is estimated following the methodology of Adrian and Brunnermeier (2016), Chun et al. (2012) and Stoyanov et al. (2013).

In Table 7, we report the three-stage least squares (3SLS) estimations. We find that GTI is positively associated with systemic risk measured by CoVaR. These results indicate high systemic risk for listed banks operating under high terrorism risk exposure.

**Table 7:** Additional analsis: using conditional value-at-risk (CoVaR) for measuring systematic risk estimation

Variables	Systemic risk
GTI	0.349^**^
	(1.86)
BSize	− 0.914^**^
	(− 2.21)
%INDEP	0.023
	(0.16)
BMEET	− 0.186
	(− 0.76)
DUAL	− 0.229^**^
	(− 2.22)
INSTITOWNER	− 0.698^***^
	(− 4.02)
ACSIZE	− 0.368
	(− 1.16)
LISTED	0.439^**^
	(2.47)
LogAge	0.611^***^
	(4.24)
LogTA	0.022
	(0.22)
TE_TA	0.000
	(1.11)
PStability	0.172
	(0.97)
IL_GL	0.009**
	(2.45)
NL_TA	0.005*
	(1.87)
GDP	− 2.138
	(− 1.03)
INFLA	− 1.696**
	(− 2.25)
Constant	− 4.218***
	(− 5.54)
Observations	648
Year Effects	YES
Country Effects	YES
R^2^	0.48
Wald Chi2	344***
LM Statistics (*p*-value)	000
Sargan test (*p*-value)	519

The table presents Three-Stage Least-Squares (3SLS) estimations for 72 listed banks identifying the impact of terrorism risk on a bank’s financial stability, which is represented by bank CoVaR for the period of 2010–2018. *P*-values in parentheses, *p < 0.10, **p < 0.05, ***p < 0.01. LM and Sargan tests show that our model is correctly identified, and our selected IVs are valid. We performed diagnostic tests (i.e., Sargan test and the Breusch and Pagan LM test) for this instrument, which show that this IV statistically satisfies the necessary conditions for validity and relevance. See Table 2 for variable definitions

Consequently, terrorist attacks increase the volatility of financial markets, which leads to high risk-taking and low financial stability for banks (see Estrada et al. 2015; Younas 2015; Arif and Suleman 2017; El Ouadghiri and Peillex 2018). Our results in Table 7 for systemic risk are consistent with the main findings and confirm our hypotheses in Table 4. Specifically, listed banks located in countries with high terrorism risk exposure are more likely to exhibit higher bank risk and low financial performance.^13^

### Robustness checks, alternative instrumental variables

To check whether our results are sensitive to the chosen exogenous IVs, we employ three additional IVs and re-estimate our main models in Eqs. (1) and (2). The first IV represents the government’s effectiveness, which reflects perceptions of the quality of public services, the quality of the civil service and the degree of its independence from political pressures, the quality of policy formulation and implementation, and the credibility of the government's commitment to such policies (source: World Bank). Therefore, we expect that terrorism risk is negatively related to government effectiveness. Secondly, we add another IV, which is the voice and accountability (VA), which reflects perceptions of the extent to which a country's citizens can participate in selecting their government, as well as freedom of expression, freedom of association, and free media (source: World Bank). We therefore expect that terrorism risk is negatively associated with the voice and accountability (VA). Finally, we employ the Fragile States Index (FSL) as an IV, which is based on the annual report published by the Fund for Peace and the American magazine Foreign Policy from 2005 to 2018. The ranking is based on the sum of scores for 12 indicators. Each indicator is scored on a scale of 0–10, with 0 being the lowest intensity (most stable) and 10 being the highest intensity (least stable), creating a scale spanning 0–120. The index tracks and assesses the performance of 178 countries on social, economic and political indicators to measure their fragility. Accordingly, we expect that terrorism risk is positively related to the control of corruption.

This instrumenting approach has been tested and applied by prior studies (e.g., Anginer et al. 2014; Safiullah and Shamsuddin 2018). An application of these instrument variables suggests that a change in the performance and/or risk of one bank is less likely to influence the terrorism risk of other banks. Hence, it is expected to be correlated with the potential endogenous variable (*GTI*) but not correlated with the dependent variables (i.e., performance and financial stability of individual banks).^14^

Our results in Table 8 for both bank risk (Panel A) and financial performance (Panel B) are consistent with the main findings in Table 4. Specifically, banks located in countries with high terrorism risk exposure are more likely to exhibit higher bank risk (i.e., high credit and insolvency risks) but they show high financial performance with low liquidity risk.

**Table 8:** Robustness check: using alternative instrument variables (IVs)

Variables	Panel A			Panel B		
	Financial risk			Financial performance		
	(1)	(2)	(3)	(4)	(5)	(6)
	LLR_GR	LA_DF	Z-score	Cost_Income	ROAA	ROAE
GTI	0.122***	0.060*	− 0.230**	− 0.053**	− 0.043	0.089*
	(0.041)	(0.035)	(0.087)	(0.026)	(0.123)	(0.052)
BSize	0.225	− 0.087	− 0.167	− 0.455***	1.532***	1.009***
	(0.209)	(0.177)	(0.448)	(0.134)	(0.472)	(0.264)
BMEET	0.244	− 0.297**	0.042	0.268**	0.440	− 0.022
	(0.158)	(0.134)	(0.338)	(0.101)	(0.354)	(0.198)
%INDEP	− 0.052	− 0.100	0.125	0.232***	− 0.154	0.036
	(0.092)	(0.078)	(0.197)	(0.059)	(0.205)	(0.114)
DUAL	− 0.087	− 0.146***	0.278**	− 0.032	0.040	0.083
	(0.058)	(0.050)	(0.125)	(0.038)	(0.131)	(0.074)
INSTITOWNER	− 0.055	− 0.060	0.311	0.031	0.027	0.247**
	(0.095)	(0.080)	(0.203)	(0.061)	(0.214)	(0.120)
ACSIZE	− 0.162	− 0.032	0.139	− 0.464***	0.473	0.354
	(0.197)	(0.167)	(0.423)	(0.126)	(0.441)	(0.247)
LogTA	− 0.027	0.023	− 0.329**	0.027	0.300**	0.115
	(0.056)	(0.047)	(0.120)	(0.036)	(0.127)	(0.070)
TE_TA	0.150***	0.308***	− 0.210**	− 0.059**	0.842***	− 0.144**
	(0.046)	(0.039)	(0.098)	(0.030)	(0.103)	(0.058)
IL_GL	0.398***	0.030*	− 0.049	0.016	− 0.241***	− 0.079***
	(0.019)	(0.016)	(0.041)	(0.012)	(0.044)	(0.024)
NL_TA	− 0.259***	− 0.323***	0.283***	− 0.060**	− 0.311***	0.034
	(0.037)	(0.032)	(0.079)	(0.024)	(0.083)	(0.046)
Cost_Income	− 0.055	− 0.108**	0.071	–	–	–
	(0.048)	(0.040)	(0.102)	–	–	–
1/Z	–	–	–	− 0.041	0.209	0.0414
	–	–	–	(0.063)	(0.220)	(0.123)
LISTED	− 0.055	− 0.091**	0.277***	0.018	− 0.173*	− 0.138**
	(0.040)	(0.034)	(0.086)	(0.026)	(0.091)	(0.051)
LogAge	0.131	0.116	0.211	0.055	0.030	0.338***
	(0.090)	(0.076)	(0.192)	(0.058)	(0.202)	(0.113)
PStability	− 0.628***	0.042	0.279	0.095*	− 0.492**	− 0.408***
	(0.084)	(0.072)	(0.181)	(0.054)	(0.201)	(0.107)
GDP	− 2.641**	0.471	0.597	− 2.531***	13.84***	5.691***
	(1.166)	(0.989)	(2.500)	(0.750)	(2.750)	(1.473)
INFLA	− 1.146***	0.210	− 0.227	− 0.022	2.406**	0.863**
	(0.322)	(0.274)	(0.692)	(0.209)	(0.758)	(0.409)
Constant	2.374***	− 3.576***	7.992***	− 7.069***	4.281***	0.164
	(0.180)	(0.620)	(0.451)	(1.280)	(0.205)	(0.526)
Observations	954	954	954	954	954	954
Year Effects	Yes	Yes	Yes	Yes	Yes	Yes
Country Effects	Yes	Yes	Yes	Yes	Yes	Yes
R^2^	0.712	0.507	0.249	0.338	0.267	0.188
Wald Chi2	432***	354***	365***	257***	490***	423***
LM Statistics (*p*-value)	000	000	000	000	000	000
Sargan test (*p*-value)	593	745	624	527	456	639

This table reports the robustness checks for main findings by employing alternative IVs (our first IV is the control of the corruption country index, our second IV for terrorism risk is the country-level income-generating category and our final IV for terrorism risk is the rule of law). LM and Sargan tests show that our models are correctly identified, and our selected IVs are valid. In both panels, control variables and year dummies are included but unreported. *P*-value in parentheses, *p < 0.10, **p < 0.05, ***p < 0.01. See Table 2 for variable definitions

### Robustness check: two-step system generalised method of moments

We check the robustness of our results using a two-step system Generalised Method of Moments (GMM) estimator (Arellano and Bover 1995; Blundell and Bond 1998). The GMM estimation procedure controls for the unobserved effects by transforming the variables into first differences to eliminate unobserved heterogeneity and omitted variable bias. It also allows us to treat all bank characteristic variables as endogenous and orthogonally employs the lag values of endogenous variables as internal IVs (Mollah et al. 2017; Abdelsalam et al. 2020). Country and macroeconomic control variables are treated as strictly exogenous. We used the lagged approach in testing our interest variable relationships using the GMM model. The results are still consistent across all our risk and performance perspectives. The validity tests confirm that our GMM estimators are valid. We report the first-order serial correlation AR (1), which shows a significant result (*p*-value < 5%) across all of our risk perspectives, indicating that the null hypothesis can be rejected and hence confirming that the residuals in the first differences are correlated. We also present the second-order correlation (AR (2)) and Hansen tests of over-identification in all our risk and performance perspectives. The AR (2) tests yield *p*-values higher than 5% for all the risk and performance measures, indicating that there is not enough evidence to reject the null hypothesis of no serial correlation of second differences. The Hansen results also show that we cannot reject the hypothesis that our instruments are valid.

Table 9, Panel A, reports the results for bank risk, and Panel B shows the results of financial performance. Our results remain consistent with the main findings in Table 4, even after controlling for unobserved heterogeneity, simultaneity and dynamic endogeneity. Table 9 also reveals the exogeneity tests of a subset of our instruments. The results show that the additional subset of instruments (as lagged differences) is exogenous.

**Table 9:** Robustness check: GMM models

Variables	Panel A			Panel B		
	Financial risk			Financial performance		
	(1)	(2)	(3)	(4)	(5)	(6)
	LLR_GR	LA_DF	Z-score	Cost_Income	ROAA	ROAE
Dependent (t_1)	0.199***	0.096	0.258**	0.620***	0.225***	0.308***
	(0.045)	(0.116)	(0.104)	(0.072)	(0.036)	(0.087)
Dependent (t_2)	0.452***	0.233**	− 0.039	− 0.127	–	–
	(0.076)	(0.083)	(0.099)	(0.095)	–	–
Dependent (t_3)	− 0.318***	− 0.074	− 0.097	0.126	–	–
	(0.070)	(0.148)	(0.083)	(0.102)	–	–
GTI	0.037**	0.059*	0.187***	0.004	0.061**	0.083**
	(0.018)	(0.035)	(0.061)	(0.010)	(0.025)	(0.033)
BSize	0.600	− 1.155*	− 4.117**	0.117	2.179**	0.264
	(0.696)	(0.692)	(1.791)	(0.330)	(0.847)	(1.154)
BMEET	0.298	− 0.050	− 0.036	0.377*	− 2.391***	− 1.454**
	(0.420)	(0.495)	(0.951)	(0.215)	(0.578)	(0.601)
%INDEP	1.499***	0.238	− 0.344*	0.498*	− 2.626***	− 0.440
	(0.433)	(0.568)	(0.908)	(0.277)	(0.788)	(0.747)
DUAL	0.059	0.076	− 1.095	− 0.017	− 0.522	− 0.602*
	(0.241)	(0.203)	(0.589)	(0.108)	(0.393)	(0.348)
INSTITOWNER	0.371	− 1.119	− 0.363	0.497	− 4.788***	− 1.709
	(0.726)	(0.707)	(1.492)	(0.433)	(1.457)	(1.269)
ACSIZE	− 1.441**	0.027	− 0.272	0.400	0.623	− 0.085
	(0.607)	(0.623)	(2.444)	(0.318)	(1.116)	(1.109)
LogTA	0.033	− 0.117	− 0.693**	0.001	− 0.107	0.001
	(0.115)	(0.137)	(0.335)	(0.057)	(0.219)	(0.220)
TE_TA	− 0.078	− 0.067	− 0.959***	− 0.143**	1.208***	0.042
	(0.128)	(0.150)	(0.273)	(0.067)	(0.287)	(0.241)
IL_GL	0.280***	0.053	− 0.090	0.014	0.074	0.001
	(0.050)	(0.050)	(0.084)	(0.024)	(0.054)	(0.056)
NL_TA	− 0.594***	− 0.524***	− 0.086	− 0.044	0.178	− 0.134
	(0.097)	(0.108)	(0.167)	(0.038)	(0.129)	(0.133)
Cost_Income	− 0.017	0.022	− 0.640***	–	–	–
	(0.069)	(0.052)	(0.215)	–	–	–
1/Z	–	–	–	0.253***	− 0.128	0.034
	–	–	–	(0.057)	(0.204)	(0.235)
LISTED	− 0.437	− 0.954	− 0.700	0.382**	− 0.487	− 1.353*
	(0.498)	(0.495)	(0.570)	(0.166)	(0.981)	(0.681)
LogAge	1.265	2.237**	− 1.043	− 0.037	0.566	1.729
	(0.823)	(0.980)	(1.416)	(0.426)	(1.625)	(1.344)
PStability	− 0.265***	0.195*	− 0.013	0.075	0.196	− 0.415**
	(0.078)	(0.100)	(0.185)	(0.053)	(0.204)	(0.183)
GDP	− 3.134***	− 1.404	− 10.04**	− 2.179**	9.943***	5.780**
	(1.069)	(1.268)	(4.627)	(0.891)	(1.779)	(2.164)
INFLA	− 2.013***	− 0.252	− 0.925	0.865***	0.889*	− 0.608
	(0.513)	(0.456)	(1.544)	(0.239)	(0.471)	(0.545)
Constant	− 0.754	− 0.823***	0.738**	0.677	0.475	1.562
	(1.898)	(0.265)	(0.166)	(0.769)	(1.369)	(2.817)
Observations	636	636	636	636	848	848
Year effects	Yes	Yes	Yes	Yes	Yes	Yes
Country effects	Yes	Yes	Yes	Yes	Yes	Yes
AR (1) test (p− value)	0.005	0.005	0.000	0.000	0.001	0.002
AR (2) test (*p*-value)	0.456	0.346	0.959	0.189	0.362	0.451
Hansen test of over-identification (*p*-value)	0.235	0.434	0.167	0.218	0.113	0.373
Diff-in- Hansen test of exogeneity (*p*-value)	0.345	0.981	0.850	0.631	0.445	0.289

The table presents regression results for terrorism risk on bank stability for full sample using GMM estimation to control for endogeneity problem. In both panels, control variables and year dummies are included but unreported. Heteroscedasticity-robust standard errors are in parentheses. *p < 0.10; **p < 0.05; ***p < 0.01. See Table 2 for variable definitions

### Robustness check: fixed effect models

In Table10, we report the fixed effect estimations as a robustness check for the main analysis applied for the full sample. Firstly, in Panel A, the GTI is positively associated with credit risk (LLR_GR) and liquidity ratio (*LA/DSF*). These findings are consistent with our main findings of bank risk and confirm our hypotheses. Secondly, the results in Panel B show that the coefficients of GTI are positively related to *COST/INCOME* but negatively associated with *ROAE*. Thus, we find low financial performance (i.e., low-cost efficiency and significantly low *ROAE* for banks).

**Table10:** Fixed effect models for the associations between terrorism risk and bank stability

Variables	Panel A			Panel B		
	Financial risk			Financial performance		
	(1)	(2)	(3)	(4)	(5)	(6)
	LLR_GR	LA_DF	Z-score	Cost_Income	ROAA	ROAE
GTI	0.016**	0.016*	0.052	0.039^**^	0.057	− 0.010**
	(1.04)	(1.63)	(1.32)	(1.76)	(1.69)	(− 1.10)
BSize	− 0.180	− 0.716^***^	− 0.147	0.677	0.539	− 0.048
	(− 0.50)	(− 3.23)	(− 0.16)	(1.31)	(0.69)	(− 0.23)
BMEET	− 0.359	0.009	1.255^*^	− 0.776^*^	− 1.032	− 0.130
	(− 1.24)	(0.05)	(1.69)	(− 1.86)	(− 1.65)	(− 0.79)
%INDEP	0.384	− 0.043	− 1.096^*^	− 0.469	− 0.838	0.087
	(1.54)	(− 0.28)	(− 1.72)	(− 1.30)	(− 1.55)	(0.61)
DUAL	0.192	0.082	0.036	0.111	0.541	− 0.206^**^
	(1.10)	(0.76)	(0.08)	(0.44)	(1.44)	(− 2.08)
INSTITOWNER	1.473^***^	− 0.416	− 1.982	0.698	− 1.176	0.457
	(2.88)	(− 1.32)	(− 1.51)	(0.95)	(− 1.06)	(1.57)
ACSIZE	0.213	− 0.317	− 1.954	− 0.769	− 0.976	0.023
	(0.45)	(− 1.09)	(− 1.62)	(− 1.13)	(− 0.95)	(0.08)
LogTA	− 0.022	0.041	− 0.626^***^	− 0.008	0.288^**^	0.007
	(− 0.36)	(1.11)	(− 4.03)	(− 0.09)	(2.19)	(0.21)
TE_TA	0.123	0.081^*^	− 0.451^**^	− 0.081	1.511^***^	0.043
	(1.60)	(1.72)	(− 2.29)	(− 0.73)	(9.05)	(0.97)
IL_GL	0.277^***^	0.011	− 0.028	− 0.064^*^	− 0.066	0.022^*^
	(11.71)	(0.77)	(− 0.47)	(− 1.87)	(− 1.29)	(1.67)
NL_TA	− 0.579^***^	− 0.451^***^	0.209	− 0.229^***^	− 0.013	0.035
	(− 9.56)	(− 12.08)	(1.34)	(− 2.63)	(− 0.10)	(1.03)
Cost_Income	− 0.029	− 0.076^**^	− 0.011	−	–	–
	(− 0.47)	(− 2.01)	(− 0.07)	−	–	–
1/Z	–	–	–	0.157	0.736	− 0.024
	–	–	–	(1.15)	(1.34)	(− 0.45)
LISTED	− 0.023	0.772^***^	− 0.904	− 0.657	− 0.634	0.086
	(− 0.06)	(3.04)	(− 0.86)	(− 1.11)	(− 0.71)	(0.37)
LogAge	0.055	0.023	0.127	0.037	0.267	0.179
	(0.213)	(1.218)	(0.627)	(0.321)	(1.349)	(2.239)
PStability	− 0.680^***^	0.050	0.543^***^	− 0.421^***^	− 0.506^***^	0.058
	(− 9.89)	(1.18)	(3.08)	(− 4.25)	(− 3.39)	(1.48)
GDP	− 1.869^*^	0.656	− 1.521	6.311^***^	13.109^***^	− 2.210^***^
	(− 1.95)	(1.11)	(− 0.62)	(4.63)	(6.37)	(− 4.10)
INFLA	− 0.963^***^	0.342^**^	− 0.744	0.930^**^	2.035^***^	0.038
	(− 3.56)	(2.05)	(− 1.07)	(2.38)	(3.46)	(0.25)
Constant	1.977^**^	5.594^***^	10.618^***^	3.337^***^	− 2.358	3.304^***^
	(2.28)	(10.45)	(4.77)	(2.74)	(− 1.29)	(6.86)
Observations	954	954	954	954	954	954
Year Effects	Yes	Yes	Yes	Yes	Yes	Yes
Country Effects	Yes	Yes	Yes	Yes	Yes	Yes
R^2^	0.38	0.23	0.09	0.13	0.22	0.16
LM Statistics (*p*-value)	0.000	0.000	0.000	0.000	0.000	0.000

The table presents fixed effect estimations for the full sample of banks identifying the impact of terrorism risk on a bank’s financial stability, which is represented by bank risk as measured through the credit risk, liquidity risk and insolvency risk (Panel A), and bank performance as measured through profitability (ROAA and ROEA), COST/INCOME ratio (Panel B). Models include a full set of control variables such as bank-level indicators, country-level indicators, and country governance indicator, but these are not reported. εit is the error term. Models are tested for the period of nine years from 2010. P-values in parentheses, *p < 0.10, **p < 0.05, ***p < 0.01. LM and Sargan tests show that our models are correctly identified, and our selected IVs are valid. We performed diagnostic tests (i.e., Sargan test and the Breusch and Pagan LM test) for this instrument, which show that this IV statistically satisfies the necessary conditions for validity and relevance. See Table 2 for variable definitions

## Conclusion

One of the most catastrophic and destroying political shocks is terrorist attacks. This paper is the first to comprehensively and jointly examine bank risk and financial performance in association with risk of terrorism. We extend global banking studies on bank stability by utilising a unique setting like the Middle East and North Africa (MENA) region, characterised by frequent terrorist attacks, vulnerable economies and substantial political unrest over the last two decades. For instance, during 2002–2018, MENA accounted globally for 36.1% of terrorist incidents, 49.3% of terrorist-induced casualties, and 21.4% of conflict deaths.

We have presented important implications for existing research and regulatory efforts to explore and identify the likely broad-based, short-run and long-run impacts on bank financial stability for vulnerable economies like MENA, while indicating signs of differential effects and systematic risk. Our results suggest an asymmetric response of banks’ risk-taking behaviour to exogenous political shocks within vulnerable economies. In particular, we find strong evidence that banks operating in these vulnerable countries exhibit an overall high-risk profile, and hence low financial stability. Moreover, our results indicate high financial performance and better cost efficiency for banks exposed to a high risk of terrorism. Additional analyses report differential impacts of risk of terrorist attacks on bank stability and performance among countries more (less) exposed to terrorism risk and across high (low) income-generating countries. In addition, our results indicate high systemic risk for the sampled banks, which are operating under high terrorism risk exposure. The overall findings are consistent with predictions and robust to alternative models and sensitivities.

The findings in this study contribute to the ongoing debate related to terrorism implications on economies as well as provide valuable policy implications for regulators and market participants engaging with banking sectors across several countries located in the MENA region. Our results show a persistent detrimental impact of terrorist attacks on bank financial stability, using various financial performance measures and risk indicators, regardless of bank location and irrespective of different bank characteristics. While policymakers and economists agree regarding a looming recession, and a possible depression across economies, the detrimental effects of such political incidents like terrorism on bank stability for some countries could be pervasive, due to the serious disruption to global supply chains, a decline in demand for imported goods and services, and a marked decrease in international tourism and business travel. Moreover, with the strategic location and oil wealth for MENA countries, the resilience of the banking sector within the region plays a pivotal role in world stability and economic prosperity.

In fact, the political violence and unrest in the MENA region had negative consequences for other regions stemming from spill over terrorism abroad, FDI losses, disrupted oil exports, reduced economic growth and large refugee flows. Many countries have already taken, or are considering, several measures to support banking resilience during/after incidents, for example, quantitative easing, direct market interventions, and fiscal stimulus and bailout packages. However, for many less-developed countries, it is not feasible to apply such policies. The international community must build up the necessary institutional infrastructure to support democracy in the region and minimise such high frequency of terrorist attacks, which have adverse implications on bank stability. We also argue that survival in the current environment of Covid-19 turmoil for banks located within vulnerable economies with a high concentration of terrorist attacks remains questionable. This study, hence, calls for coordinated global responses to support the banking industry, which could be considered among countries located within the same region. A lack of coordination might affect different market participants and stakeholders, who could struggle to engage with their banks in the long term, and who might lose public trust with regard to the whole intermediation system. The findings are also important to depositors engaging with the banking sectors across different economies (i.e., high versus low income generating) during this stressful period of attacks, and to bank managers seeking to identify the key drivers of bank financial stability in the long term. By gaining a perspective on different indicators of risk and financial performance in relation to terrorism as a key driver of bank stability, the global community is better able to decide how best to mitigate instabilities in the MENA banking sector as well as minimise any associated transitional effects on international financial markets.

Moreover, future studies could benefit from our study by including other countries and other measures of financial stability that provide additional valuable insights to this line of literature, e.g., examining alternative banking systems (i.e., Islamic versus conventional banks). Finally, we have adopted quantitative research methods in this research; however, examining the perceptions of financial firms’ managers and directors through the application of qualitative methods could provide interesting and in-depth insights into our understanding of the link between terrorism and bank financial stability. All these avenues are therefore left to future research.

### Electronic supplementary material

Below is the link to the electronic supplementary material.

Supplementary file1 (DOCX 38 kb)

## Appendix A

See Table

**Table 11:** The Middle East and North Africa GTI Rank 2002–2018

Country	OVERALL SCORE	OVERALL Rank	CHANGE 2002–2018	CHANGE 2017–2018
Iraq	9.241	2	5.535	− 0.505
Syria	8.006	4	7.996	− 0.309
Yemen	7.259	8	4.391	− 0.275
Egypt	6.794	11	6.417	− 0.551
Libya	6.766	12	6.766	− 0.221
Sudan	5.807	20	− 0.757	− 0.371
Saudi Arabia	5.238	30	3.233	− 0.241
Palestine	5.177	32	− 0.869	− 0.153
Iran	4.717	39	2.423	0.318
Israel	4.525	40	− 2.265	− 0.053
Lebanon	4.395	43	1.178	− 0.759
Tunisia	3.938	51	0.359	− 0.150
Algeria	3.409	57	− 3.754	− 0.354
Bahrain	3.201	61	3.201	− 0.682
Jordan	3.091	64	1.074	− 0.313
Kuwait	2.487	75	2.143	− 0.639
Morocco	1.215	92	1.215	1.177
United Arab Emirates	0.048	130	0.048	− 0.057
Qatar	0.029	133	0.029	− 0.028
Oman	0.000	138	0.000	0.000
	Regional	Average	1.918	− 0.208

The Global Terrorism Index (GTI) is a comprehensive measure analysing the impact of terrorism for 163 countries and covers 99.7% of the world’s population. The index is produced by the Institute for Economics and Peace (IEP) based on data from the Global Terrorism Database (GTD) collected by the National Consortium for the Study of Terrorism and Responses to Terrorism (START) at the University of Maryland. The GTD contains over 170,000 terrorist incidents for the period 1970–2018

^11^.

## Footnotes

1 Terrorism is defined as the premeditated use of violence by individuals or sub-national groups to obtain a political or social objective through the intimidation of a large audience beyond that of the immediate victims (Enders and Sandler 2011).

2 The political turmoil was started by the successful uprising in Tunisia, which emboldened similar protests in many Middle Eastern countries. Several MENA countries are still undergoing political transitions, such as Egypt and Tunisia, alongside the political instability and civil wars in countries like Iraq, Yemen, Syria and Libya. The oil-rich monarchies of the Gulf countries remained largely unaffected by this geo-political event. However, the tensions between Saudi Arabia, the UAE and Iran, founded on religious and ideological differences, have promoted serious conflicts throughout the region (Kim and Sandler 2020; Bardwell and Iqbal 2021).

3 We would like to thank the anonymous reviewer for suggesting using CoVaR as an additional financial stability measure.

4 The variation in global terrorism index (GTI) score for the MENA region is reported in Appendix A.

5 This fundamental risk–return relation is documented in the financial literature (see for details: Belkhir et al. 2019).

6 For robustness checks, we use a three-year and five-year rolling average of standard deviation and re-calculate our *Z-score* (Trinh et al. 2020a, b). However, our results are not sensitive to this change.

7 We performed the Wu-Hausman endogeneity test across all our test models to examine whether endogeneity exists. The test statistics suggest the presence of endogeneity bias.

8 Middle- and high-income nations are classified by the World Bank (2015).

9 We performed two diagnostic tests to identify the validity of the IVs and the specification of our system equations, the Sargan test and the Breusch and Pagan LM test. Both IVs theoretically and statistically satisfy the necessary conditions for validity and relevance, and hence 3SLS results tend to be consistent and more efficient than OLS.

10 A country is classified as a high terrorism risk country (HTRc) if its GTI score is equal to or higher than the mean of the Global Terrorism Index (GTI), while a lower terrorism risk country (LTRc) has a GIT score equal to or lower than the mean of this index.

11 The study’s correlation matrix is presented within a supplementary file to this paper, for the sake of brevity.

12 The income classification is based on a measure of national income per person, or *GNI* per capita, calculated using the Atlas method. As of 1 July 2019, low-income economies are defined as those with a *GNI* per capita of $1,025 or less in 2018; lower-middle-income economies are those with a *GNI* per capita between $1,026 and $3,995; upper-middle-income economies are those between $3,996 and $12,375; and high-income economies are those with a *GNI* per capita of $12,376 or more.

13 We additionally examine the impact of risk of terrorism on bank stability while identifying the effect of banks' life cycle. Our main findings remain unchanged, and results indicate that terrorism increases bank risk for young banks whereas mature banks can mitigate various types of bank risk. Our findings are presented in Appendix 2, within a supplementary file to this paper, for the sake of brevity.

14 We performed two diagnostic tests to identify the validity of the IVs and the specification of our system equations, the Sargan test and the Breusch and Pagan LM test. All these IVs theoretically and statistically satisfy the necessary conditions for validity and relevance, and hence 3SLS results tend to be consistent and more efficient than OLS.

## References

[CR1] Abadie A, Gardeazabal J (2003). The economic costs of conflict: a case study of the Basque country. Am Econ Rev.

[CR2] Abadie A, Gardeazabal J (2008). Terrorism and the world economy. Eur Econ Rev.

[CR3] Abedifar P, Molyneux P, Tarazi A (2013). Risk in Islamic banking. Rev Financ.

[CR4] Abdallah AAN, Ismail AK (2017). Corporate governance practices, ownership structure, and corporate performance in the GCC countries. J Int Finan Markets Inst Money.

[CR5] Abdelsalam O, Elnahass M, Ahmed H, Williams J (2020). Asset securitizations and bank stability: evidence from different banking systems. Glob Financ J.

[CR6] Adrian T, Brunnermeier MK (2016). CoVaR. Am Econ Rev.

[CR7] Alam A (2013). Terrorism and stock market development: causality evidence from Pakistan. J Financ Crime.

[CR9] Anginer D, Demirguc-Kunt A, Zhu M (2014). How does competition affect bank systemic risk?. J Financ Intermed.

[CR10] Arellano M, Bover O (1995). Another look at the instrumental variable estimation of error-components models. J Econom.

[CR11] Arif I, Suleman T (2017). Terrorism and stock market linkages: an empirical study from a front-line state. Glob Bus Rev.

[CR12] Arin KP, Ciferri D, Spagnolo N (2008). The price of terror: the effects of terrorism on stock market returns and volatility. Econ Lett.

[CR13] Arnaboldi F, Casu B, Kalotychou E, Sarkisyan A (2020). The performance effects of board heterogeneity: what works for EU banks?. Eur J Financ.

[CR14] Ashraf BN (2017). Political institutions and bank risk-taking behavior. J Financ Stab.

[CR16] Asongu SA, Nwachukwu JC (2017). Revolution empirics: predicting the Arab Spring. Empir Econ.

[CR17] Asongu SA, Nwachukwu JC (2017). The impact of terrorism on governance in African countries. World Dev.

[CR18] Bandyopadhyay S, Sandler T, Younas J (2014). Foreign direct investment, aid, and terrorism. Oxf Econ Pap.

[CR19] Bardwell H, Iqbal M (2021). The economic impact of terrorism from 2000 to 2018. Peace Econ Peace Sci Public Policy.

[CR20] Belghitar Y, Clark E, Saeed A (2019). Political connections and corporate financial decision making. Rev Quant Financ Acc.

[CR21] Belkhir M, Grira J, Hassan M, Soumaré I (2019). Islamic banks and political risk: international evidence. Q Rev Econ Financ.

[CR22] Bitar M, Hassan MK, Walker T (2017). Political systems and the financial soundness of Islamic banks. J Financ Stab.

[CR23] Bitar M, Pukthuanthong K, Walker T (2018). The effect of capital ratios on the risk, efficiency and profitability of banks: evidence from OECD countries. J Int Finan Markets Inst Money.

[CR24] Blomberg SB, Hess GD, Weerapana A (2004). Economic conditions and terrorism. Eur J Polit Econ.

[CR25] Boateng A, Huang W, Kufuor NK (2015). Commercial bank ownership and performance in China. Appl Econ.

[CR26] Borri N, Caccavaio M, Giorgio GD, Sorrentino AM (2014). Systemic risk in the Italian banking industry. Econ Notes Rev Bank Financ Monet Econ.

[CR27] Boussiga N, Ghdamsi M (2016). The corruption-terrorism nexus: a panel data approach. Int J Econ Financ.

[CR28] Brewer TL, Rivoli P (1990). Politics and perceived country creditworthiness in international banking. J Money Credit Bank.

[CR29] Buch CM, Eickmeier S, Prieto E (2014). Macroeconomic factors and microlevel bank behavior. J Money Credit Bank.

[CR30] Blundell R, Bond S (1998). Initial conditions and moment restrictions in dynamic panel data models. J Econom.

[CR32] Caporaso JA, Levine DP (1992). Theories of political economy.

[CR33] Caprio G, Honohan P (1999). Restoring banking stability: beyond supervised capital requirements. J Econ Perspect.

[CR34] Chan KK, Milne A (2014). Bank competition, fire-sales and financial stability. Eur J Financ.

[CR35] Chen AH, Siems TF (2004). The effects of terrorism on global capital markets. Eur J Polit Econ.

[CR36] Chesney M, Reshetar G, Karaman M (2011). The impact of terrorism on financial markets: an empirical study. J Bank Financ.

[CR37] Choi SW (2010). Fighting terrorism through the rule of law?. J Conflict Resolut.

[CR38] Chun SY, Shapiro A, Uryasev S (2012). Conditional value-at-risk and average value-at-risk: estimation and asymptotics. Oper Res.

[CR39] Čihák M, Hesse H (2010). Islamic banks and financial stability: an empirical analysis. J Financ Serv Res.

[CR8] De Andres P, Vallelado E (2008). Corporate governance in banking: the role of the board of directors. J Bank Financ.

[CR40] De Mendonça HF, Da Silva RB (2018). Effect of banking and macroeconomic variables on systemic risk: an application of ΔCOVAR for an emerging economy. N Am J Econ Financ.

[CR41] De Mesquita EB (2005). The quality of terror. Am J Polit Sci.

[CR42] Demirgüç-Kunt A (1989). Deposit-institution failures: a review of empirical literature. Econ Rev.

[CR43] Demirgüç-Kunt A, Detragiache E (1998). The determinants of banking crises in developing and developed countries. IMF Econ Rev.

[CR122] De Vita G, Trachanas E, Luo Y (2018). Revisiting the bi-directional causality between debt and growth: evidence from linear and nonlinear tests. J Int Money Financ.

[CR44] Ding D, Sickles RC (2018). Frontier efficiency, capital structure, and portfolio risk: an empirical analysis of US banks. BRQ Bus Res Q.

[CR45] Dong Y, Girardone C, Kuo JM (2017). Governance, efficiency and risk taking in Chinese banking. Br Account Rev.

[CR46] Drakos K, Gofas A (2004) The determinants of terrorist activity: a simple model for attack occurrence across space and time. In: Paper prepared for conference on the Political Economy of Terrorism. University of Southern California

[CR47] Drakos K (2010). Terrorism activity, investor sentiment, and stock returns. Rev Financ Econ.

[CR48] Efobi U, Asongu S (2016). Terrorism and capital flight from Africa. Int Econ.

[CR49] Efobi U, Asongu S, Beecroft I (2015) Foreign direct investment, aid and terrorism: empirical insight conditioned on corruption control. Research Africa Network Working Papers 15/007, Research Africa Network (RAN)

[CR50] El Ouadghiri I, Peillex J (2018). Public attention to “Islamic terrorism” and stock market returns. J Comp Econ.

[CR51] Eldor R, Melnick R (2004). Financial markets and terrorism. Eur J Polit Econ.

[CR52] Elnahass M, Izzeldin M, Steele G (2018). Capital and earnings management: evidence from alternative banking business models. Int J Account.

[CR53] Elnahass M, Omoteso K, Salama A, Trinh VQ (2020). Differential market valuations of board busyness across alternative banking models. Rev Quant Financ Acc.

[CR54] Elnahass M, Salama A, Trinh VQ (2020). Firm valuations and board compensation: evidence from alternative banking models. Glob Financ J.

[CR55] Elnahass M, Trinh VQ, Li T (2021). Global banking stability in the shadow of Covid-19 outbreak. J Int Financ Markets Inst Money.

[CR56] Elyasiani E, Zhang L (2015). Bank holding company performance, risk, and “busy” board of directors. J Bank Financ.

[CR57] Enders W, Sandler T (2011). The political economy of terrorism.

[CR58] Essaddam N, Karagianis JM (2014). Terrorism, country attributes, and the volatility of stock returns. Res Int Bus Financ.

[CR59] Estrada MAR, Park D, Kim JS, Khan A (2015). The economic impact of terrorism: a new model and its application to Pakistan. J Policy Model.

[CR60] Faleye O, Krishnan K (2017). Risky lending: does bank corporate governance matter?. J Bank Financ.

[CR61] Fernández AI, González F, Suárez N (2016). Banking stability, competition, and economic volatility. J Financ Stab.

[CR62] Ferris SP, Jagannathan M, Pritchard AC (2003). Too busy to mind the business? Monitoring by directors with multiple board appointments. J Financ.

[CR63] Fosu S, Danso A, Agyei-Boapeah H, Ntim CG, Adegbite E (2020). Credit information sharing and loan default in developing countries: the moderating effect of banking market concentration and national governance quality. Rev Quant Financ Acc.

[CR64] Frey BS (2004). Dealing with terrorism: stick or carrot?.

[CR66] Gasbarro D, Sadguna IGM, Zumwalt JK (2002). The changing relationship between CAMEL ratings and bank soundness during the Indonesian banking crisis. Rev Quant Financ Acc.

[CR67] Graham MA, Ramiah VB (2012). Global terrorism and adaptive expectations in financial markets: evidence from Japanese equity market. Res Int Bus Financ.

[CR68] Hanif H, Naveed M, Rehman MU (2019). Dynamic modeling of systemic risk and firm value: a case of Pakistan. Cogent Bus Manag.

[CR69] Heffernan S, Fu M (2008) The determinants of bank performance in China. Available at SSRN 1247713.

[CR70] Howarth D, Quaglia L (2013). Banking on stability: the political economy of new capital requirements in the European Union. J Eur Integr.

[CR71] Huang Q, De Haan J, Scholtens B (2019). Analysing systemic risk in the Chinese banking system. Pac Econ Rev.

[CR72] Hughes JP, Mester LJ, Moon CG (2001). Are scale economies in banking elusive or illusive?: Evidence obtained by incorporating capital structure and risk-taking into models of bank production. J Bank Financ.

[CR73] Imerman MB (2020). When enough is not enough: bank capital and the Too-Big-To-Fail subsidy. Rev Quant Financ Acc.

[CR74] Jiang H, Zhang J, Sun C (2020). How does capital buffer affect bank risk-taking? New evidence from China using quantile regression. China Econ Rev.

[CR75] Johnston RB, Nedelescu OM (2006). The impact of terrorism on financial markets. J Financ Crime.

[CR76] Jokipii T, Monnin P (2013). The impact of banking sector stability on the real economy. J Int Money Financ.

[CR77] Karshenas M, Moghadam VM, Alami R (2014). Social policy after the Arab spring: states and social rights in the MENA region. World Dev.

[CR78] Khandelwal P, Roitman A (2013) The economics of political transitions: implications for the Arab Spring. International Monetary Fund. IMF Working Paper WP/13/69

[CR79] Kim D, Sohn W (2017). The effect of bank capital on lending: does liquidity matter?. J Bank Financ.

[CR80] Kim W, Sandler T (2020). Middle East and North Africa: terrorism and conflicts. Global Pol.

[CR81] Kollias C, Kyrtsou C, Papadamou S (2013). The effects of terrorism and war on the oil price–stock index relationship. Energy Econ.

[CR82] Kollias C, Papadamou S, Stagiannis A (2011). Terrorism and capital markets: the effects of the Madrid and London bomb attacks. Int Rev Econ Financ.

[CR83] Kress JC (2018). Board to death: how busy directors could cause the next financial crisis. Boston Coll Law Rev.

[CR84] Liang Q, Xu P, Jiraporn P (2013). Board characteristics and Chinese bank performance. J Bank Financ.

[CR85] Larobina MD, Pate RL (2009). The impact of terrorism on business. J Global Bus Issues.

[CR86] Lee CC, Hsieh MF (2013). The impact of bank capital on profitability and risk in Asian banking. J Int Money Financ.

[CR87] Lenain P, Bonturi M, Koen V (2002) The economic consequences of terrorism (OECD Economics Department Working Papers, No. 334). Paris: OECD Publishing

[CR88] Liargovas P, Repousis S (2010). The impact of terrorism on Greek banks’ stocks: an event study. Int Res J Financ Econ.

[CR89] Liu WM, Ngo PT (2014). Elections, political competition and bank failure. J Financ Econ.

[CR90] Macdonald S, Correia SG, Watkin AL (2019). Regulating terrorist content on social media: automation and the rule of law. Int J Law Context.

[CR91] Marie M, Kamel H, Elbendary I (2021). How does internal governance affect banks’ financial stability? Empirical evidence from Egypt. Int J Discl Gov.

[CR92] Mester LJ, Thakor A, Boot WA (2008). Optimal industrial structure in banking. Handbooks in finance, handbook of financial intermediation and banking.

[CR93] Mollah S, Zaman M (2015). Shari’ah supervision, corporate governance and performance: conventional vs. Islamic banks. J Bank Financ.

[CR94] Mollah S, Hassan MK, Al Farooque O, Mobarek A (2017). The governance, risk-taking, and performance of Islamic banks. J Financ Serv Res.

[CR95] Neuman GL (2004). Comment, counter-terrorist operations and the rule of law. Eur J Int Law.

[CR96] Ozili PK (2020). Bank loan loss provisioning during election years: cross-country evidence. Int J Manag Financ.

[CR97] Ozili PK (2018). Banking stability determinants in Africa. Int J Manag Financ.

[CR98] Ozili PK (2019). Non-performing loans in European systemic and non-systemic banks. J Financ Econ Policy.

[CR99] Ozili PK (2019). Non-performing loans and financial development: new evidence. J Risk Financ.

[CR100] Peleg K, Regens JL, Gunter JT, Jaffe DH (2011). The normalisation of terror: the response of Israel's stock market to long periods of terrorism. Disasters.

[CR101] Rahman MA, Hassan MK (2013). Firm fundamentals and stock prices in emerging Asian stock markets: some panel data evidence. Rev Quant Financ Acc.

[CR102] Ramiah V, Graham M (2013). The impact of domestic and international terrorism on equity markets: evidence from Indonesia. Int J Account Inf Manag.

[CR103] Raza SA, Jawaid ST (2013). Terrorism and tourism: a conjunction and ramification in Pakistan. Econ Model.

[CR104] Roengpitya R, Rungcharoenkitkul P (2011). Measuring systemic risk and financial linkages in the Thai banking system. Syst Risk Basel III Financ Stab Regul.

[CR105] Rumler F, Waschiczek W (2016). Have changes in the financial structure affected bank profitability? Evidence for Austria. Eur J Financ.

[CR106] Safiullah M, Shamsuddin A (2018). Risk in Islamic banking and corporate governance. Pac Basin Financ J.

[CR107] Sandler T, Enders W (2008). Economic consequences of terrorism in developed and developing countries: an overview. Terror Econ Dev Polit Open.

[CR108] Sandler T, George J (2016). Military expenditure trends for 1960–2014 and what they reveal. Global Pol.

[CR109] Segoviano Basurto M, Goodhart C (2009) Banking stability measures. IMF working papers 1–54.

[CR110] Shahbaz M (2013). Linkages between inflation, economic growth and terrorism in Pakistan. Econ Model.

[CR111] Shelley L (2004). The unholy trinity: transnational crime, corruption, and terrorism. Brown J World Affairs.

[CR112] Shrieves RE, Dahl D (1992). The relationship between risk and capital in commercial banks. J Bank Financ.

[CR113] Simpson M (2014). Terrorism and corruption: alternatives for goal attainment within political opportunity structures. Int J Sociol.

[CR114] Simser J (2011). Terrorism financing and the threat to financial institutions. J Money Laund Control.

[CR116] Stolbov M (2017). Assessing systemic risk and its determinants for advanced and major emerging economies: the case of ΔCoVaR. IEEP.

[CR117] Stoyanov SV, Rachev ST, Fabozzi FJ (2013). CVaR sensitivity with respect to tail thickness. J Bank Financ.

[CR118] Su S, Ahmad AH, Wood J (2019). How effective is central bank communication in emerging economies? An empirical analysis of the Chinese money markets responses to the people’s bank of China’s policy communications. Rev Quant Financ Acc.

[CR120] Trinh VQ, Elnahass M, Salama A (2020). Board busyness and new insights into alternative bank dividends models. Rev Quant Financ Acc.

[CR121] Trinh VQ, Elnahass M, Salama A, Izzeldin M (2020). Board busyness, performance and financial stability: does bank type matter?. Eur J Financ.

[CR123] World Bank (2015) World Bank country and lending groups. https://datahelpdesk.worldbank.org. Accessed 02 July 2020

[CR124] World Bank (2016) Labor market polarization in developing countries: challenges ahead. http://blogs.worldbank.org. Accessed 02 July 2020

[CR125] Younas J (2015). Terrorism, openness and the Feldstein-Horioka paradox. Eur J Polit Econ.

