# Supplementary material for: Terrorist attacks and bank financial stability: evidence from MENA economies
Source: Rev Quant Finan Acc. 2022 Feb 19;59(1):383–427. doi: 10.1007/s11156-022-01043-1 (PMC8857896; doi:10.1007/s11156-022-01043-1)
Supplement: Supplementary file 1 — Supplementary file1 (DOCX 38 kb) [file 11156_2022_1043_MOESM1_ESM.docx]

**Appendices**

**Appendix 1:** Appendix B presents the Pearson correlation coefficients of all dependent and independent variables for the full sample. All correlations are in line with expectations and the matrix of the correlation coefficients affirms that multicollinearity does not appear to be a serious statistical problem. This is also supported by the unreported low individual VIF values (< 10), low means of VIFs (< 6), and low condition numbers (< 15) for all the test variables that are showing no multicollinearity problems among the regressors.

| **Appendix B. Pearson Pairwise Correlation Matrix: Full Sample** | | | | | | | | | | | | |
| --- | --- | --- | --- | --- | --- | --- | --- | --- | --- | --- | --- | --- |
| **Variables** | **(1)** | **(2)** | **(3)** | **(4)** | **(5)** | **(6)** | **(7)** | **(8)** | **(9)** | **(10)** | **(11)** | **(12)** |
| **(1) LLR_GR** | 1.000 |  |  |  |  |  |  |  |  |  |  |  |
| **(2) LA_DF** | 0.35* | 1.000 |  |  |  |  |  |  |  |  |  |  |
| **(3) Z-score** | -0.27* | -0.22* | 1.000 |  |  |  |  |  |  |  |  |  |
| **(4) Cost_Income** | -0.10* | -0.06* | 0.11* | 1.000 |  |  |  |  |  |  |  |  |
| **(5) ROAA** | -0.01 | 0.13* | -0.13* | -0.50* | 1.000 |  |  |  |  |  |  |  |
| **(6) ROAE** | -0.04 | 0.01 | -0.15* | -0.43* | 0.54* | 1.000 |  |  |  |  |  |  |
| **(7) GTI** | 0.21* | 0.29* | -0.17* | 0.15* | -0.02 | 0.16* | 1.000 |  |  |  |  |  |
| **(8) BSize** | -0.23* | -0.26* | 0.18* | 0.08* | -0.06* | 0.04 | 0.05 | 1.000 |  |  |  |  |
| **(9) BMEET** | -0.16* | -0.28* | 0.17* | 0.21* | -0.17* | -0.05 | -0.01 | 0.25* | 1.000 |  |  |  |
| **(10) %INDEP** | 0.06 | 0.07* | -0.13* | -0.03 | 0.03 | 0.09* | -0.02 | -0.14* | 0.08* | 1.000 |  |  |
| **(11) DUAL** | -0.12* | 0.02 | 0.02 | 0.01 | -0.02 | 0.11* | 0.19* | -0.03 | -0.11* | 0.02 | 1.000 |  |
| **(12) INSTITOWNER** | -0.07* | 0.09* | -0.05 | 0.06* | 0.05 | 0.05 | 0.05 | -0.14* | -0.24* | -0.15* | 0.01 | 1.000 |
| **(13) ACSIZE** | -0.23* | -0.22* | 0.21* | 0.01 | -0.01 | 0.05 | -0.09* | 0.31* | 0.23* | -0.09* | 0.12* | 0.08* |
| **(14) LogTA** | -0.02 | -0.05 | -0.09* | -0.01 | 0.09* | 0.11* | -0.01 | 0.12* | 0.03 | -0.04 | 0.17* | -0.02 |
| **(15) TE_TA** | 0.22* | 0.33* | -0.06 | -0.24* | 0.28* | -0.18* | -0.30* | -0.26* | -0.40* | -0.01 | -0.13* | 0.09* |
| **(16) IL_GL** | 0.72* | 0.28* | -0.20* | 0.02 | -0.05 | -0.09* | 0.16* | -0.15* | -0.17* | 0.02 | -0.13* | -0.04 |
| **(17) NL_TA** | -0.44* | -0.52* | 0.21* | -0.15* | -0.07* | 0.06 | -0.38* | 0.27* | 0.16* | -0.02 | 0.23* | -0.02 |
| **(18) LISTED** | -0.04 | -0.22* | 0.20* | -0.13* | -0.01 | -0.07* | -0.24* | 0.29* | 0.00 | -0.10* | 0.20* | -0.16* |
| **(19) LogAge** | -0.31* | -0.36* | 0.15* | -0.25* | 0.10* | -0.04 | -0.86* | -0.04 | 0.05 | 0.07* | 0.08* | -0.05 |
| **(20) PStability** | -0.14* | -0.07* | 0.05 | 0.14* | -0.14* | 0.08* | 0.15* | 0.14* | 0.22* | 0.01 | 0.06 | 0.10* |
| **(21) GDP** | -0.12* | 0.00 | -0.03 | -0.08* | 0.15* | 0.10* | -0.09* | -0.09* | 0.11* | 0.13* | 0.02 | -0.02 |
| **(22) INFLA** | 0.17* | 0.09* | -0.29* | 0.04 | 0.02 | 0.11* | 0.27* | -0.03 | -0.02 | 0.02 | -0.08* | -0.02 |
|  | **(13)** | **(14)** | **(15)** | **(16)** | **(17)** | **(18)** | **(19)** | **(20)** | **(21)** | **(22)** |  |  |
| **(13) ACSIZE** | 1.000 |  |  |  |  |  |  |  |  |  |  |  |
| **(14) LogTA** | 0.17* | 1.000 |  |  |  |  |  |  |  |  |  |  |
| **(15) TE_TA** | -0.13* | -0.09* | 1.000 |  |  |  |  |  |  |  |  |  |
| **(16) IL_GL** | -0.13* | 0.00 | 0.16* | 1.000 |  |  |  |  |  |  |  |  |
| **(17) NL_TA** | 0.23* | 0.10* | -0.10* | -0.38* | 1.000 |  |  |  |  |  |  |  |
| **(18) LISTED** | 0.20* | 0.05 | 0.07* | -0.00 | 0.19* | 1.000 |  |  |  |  |  |  |
| **(19) LogAge** | 0.06 | -0.03 | -0.32* | -0.17* | 0.01 | -0.24* | 1.000 |  |  |  |  |  |
| **(20) PStability** | 0.08* | 0.04 | 0.22* | -0.30* | 0.50* | 0.21* | -0.11* | 1.000 |  |  |  |  |
| **(21) GDP** | 0.02 | 0.05 | 0.02 | -0.07* | 0.07* | -0.03 | -0.04 | 0.20* | 1.000 |  |  |  |
| **(22) INFLA** | -0.08* | 0.01 | -0.17* | 0.23* | -0.16* | -0.09* | 0.04 | -0.33* | 0.06 | 1.000 |  |  |

Notes: The table presents pairwise correlation between all variables used in all the regression models for the full sample. The table reveals the absence of a high correlation between the key variables. The variance inflation factors (VIFs) also show no multicollinearity problems among the regressors. There is no coefficient value higher than 80% between the explanatory variables, as the pairwise test shows. Also, there is no VIF value of each variable that has a value high. *shows significance at the 0.05 level. See Table 2 for variable definition.

**Appendix 2: Tests for the bank’s life cycle (Age)**

We additionally examine the impact of risk of terrorism on bank stability while identifying the effect of banks' life cycle.^[[1]](#footnote-1)^ A bank is classified as a mature bank (older life cycle) if its age is equal to or higher than the mean of banks’ age (i.e., 11 years), whilst it is classified as a growing bank (younger life cycle) if its age is lower than the mean. The results in Panel A in Appendix C show that terrorism does not significantly affect the overall financial stability for mature banks. In contrast, the results of younger banks in Panel B report significantly high credit risk (LLR_GR) and high insolvency risk (Z-score) but low liquidity risk (LA/DSF). Moreover, the GTI is significantly and positively associated with ROAE, in line with modern portfolio theory for risk-return. These findings are consistent with our main findings and confirm our hypotheses. These results indicate that terrorism increases bank risk for young banks whereas mature banks can mitigate various types of bank risk. These results are consistent with DeYoung and Hasan (1998), who provide evidence that the performance of mature banks is more resilient and stronger than that of young banks.

| **Variables** | **Appendix C. Terrorism Risk and Bank Stability: Banks’ Life Cycle** | | | | | | | | | | | |
| --- | --- | --- | --- | --- | --- | --- | --- | --- | --- | --- | --- | --- |
|  | **Mature Banks** | | | | | | **Young Banks** | | | | | |
|  | **Panel A** | | | | | | **Panel B** | | | | | |
|  | **Financial Risk** | | | **Financial Performance** | | | **Financial Risk** | | | **Financial Performance** | | |
|  | **(1)** | **(2)** | **(3)** | **(4)** | **(5)** | **(6)** | **(7)** | **(8)** | **(9)** | **(10)** | **(11)** | **(12)** |
|  | **LLR_GR** | **LA_DF** | **Z-score** | **Cost_Income** | **ROAA** | **ROAE** | **LLR_GR** | **LA_DF** | **Z-score** | **Cost_Income** | **ROAA** | **ROAE** |
| **GTI** | -0.064 | -0.046 | -0.305* | -0.033 | 0.184* | 0.156* | 0.132*** | 0.096** | -0.262*** | 0.054 | -0.047 | 0.141** |
|  | (0.060) | (0.054) | (0.178) | (0.038) | (0.112) | (0.086) | (0.043) | (0.036) | (0.073) | (0.034) | (0.114) | (0.065) |
| **BSize** | -0.197 | -1.088*** | -1.674** | -0.761*** | 0.763 | 1.196*** | 0.394 | -0.042 | 0.613 | -0.183 | 1.963*** | 0.759* |
|  | (0.323) | (0.294) | (0.829) | (0.173) | (0.572) | (0.394) | (0.275) | (0.209) | (0.462) | (0.204) | (0.662) | (0.392) |
| **BMEET** | 0.271 | -0.552** | -0.280 | 0.122 | 1.214*** | 0.007 | 0.078 | -0.148 | 0.040 | 0.229 | -0.224 | -0.084 |
|  | (0.215) | (0.195) | (0.551) | (0.116) | (0.391) | (0.264) | (0.253) | (0.193) | (0.424) | (0.188) | (0.609) | (0.361) |
| **%INDEP** | -0.160 | -0.124 | 0.689** | 0.276*** | 0.150 | 0.212 | 0.087 | 0.165 | -0.476* | 0.662*** | -1.347*** | -0.271 |
|  | (0.127) | (0.115) | (0.324) | (0.067) | (0.224) | (0.152) | (0.160) | (0.122) | (0.271) | (0.115) | (0.373) | (0.221) |
| **DUAL** | -0.135 | -0.143** | 0.386** | -0.045 | 0.020 | -0.005 | -0.109 | -0.006 | -0.196 | -0.055 | 0.516 | 0.139 |
|  | (0.071) | (0.065) | (0.182) | (0.038) | (0.128) | (0.087) | (0.141) | (0.107) | (0.236) | (0.104) | (0.338) | (0.201) |
| **INSTITOWNER** | -0.096 | -0.206* | 1.055*** | 0.096 | 0.271 | 0.575*** | -0.010 | 0.089 | -0.384 | 0.104 | -0.116 | 0.020 |
|  | (0.125) | (0.113) | (0.319) | (0.067) | (0.225) | (0.154) | (0.145) | (0.111) | (0.245) | (0.108) | (0.349) | (0.208) |
| **ACSIZE** | 0.516 | -0.261 | 1.647** | -1.022*** | -0.530 | 0.632* | -0.121 | 0.171 | -0.407 | 0.668** | 0.076 | -0.362 |
|  | (0.289) | (0.263) | (0.743) | (0.150) | (0.498) | (0.340) | (0.319) | (0.243) | (0.535) | (0.236) | (0.761) | (0.453) |
| **LogTA** | -0.125 | -0.015 | -0.478** | 0.122*** | 0.184 | 0.163* | 0.006 | 0.160** | -0.233 | -0.038 | 0.552** | 0.098 |
|  | (0.073) | (0.066) | (0.186) | (0.039) | (0.130) | (0.088) | (0.088) | (0.067) | (0.149) | (0.066) | (0.212) | (0.126) |
| **TE_TA** | 0.174** | 0.610*** | -0.237 | -0.305*** | 0.582*** | 0.032 | 0.145** | 0.171*** | -0.253** | 0.174*** | 1.073*** | -0.377*** |
|  | (0.073) | (0.066) | (0.187) | (0.037) | (0.125) | (0.084) | (0.070) | (0.053) | (0.118) | (0.051) | (0.166) | (0.099) |
| **IL_GL** | 0.378*** | 0.014 | -0.082 | 0.007 | -0.184*** | -0.042 | 0.386*** | 0.033 | -0.002 | 0.006 | -0.151** | -0.091** |
|  | (0.025) | (0.023) | (0.066) | (0.014) | (0.047) | (0.032) | (0.029) | (0.022) | (0.049) | (0.022) | (0.070) | (0.041) |
| **NL_TA** | -0.306*** | -0.707*** | 0.464** | 0.114** | -0.090 | 0.032 | -0.258*** | -0.302*** | 0.119 | -0.088** | -0.348*** | -0.002 |
|  | (0.088) | (0.080) | (0.224) | (0.047) | (0.157) | (0.107) | (0.043) | (0.033) | (0.073) | (0.032) | (0.103) | (0.061) |
| **Cost_Income** | 0.066 | 0.020 | 0.111 | - | - | - | -0.108* | -0.157*** | 0.115 | - | - | - |
|  | (0.080) | (0.073) | (0.205) | - | - | - | (0.063) | (0.048) | (0.105) | - | - | - |
| **1/Z** | - | - | - | -0.105* | 0.084 | 0.218 | - | - | - | -0.024 | 0.387 | -0.120 |
|  | - | - | - | (0.063) | (0.274) | (0.143) | - | - | - | (0.114) | (0.239) | (0.220) |
| **LISTED** | -0.151** | -0.143*** | 0.430*** | 0.024 | -0.180* | -0.013 | 0.018 | -0.063 | 0.010 | 0.088** | -0.389** | -0.332*** |
|  | (0.054) | (0.049) | (0.138) | (0.030) | (0.098) | (0.067) | (0.060) | (0.046) | (0.100) | (0.045) | (0.144) | (0.086) |
| **LogAge** | 0.468** | 0.064 | 0.933* | -0.370*** | -0.909** | 0.509** | -0.367** | -0.116 | 0.418 | -0.011 | 1.414*** | 0.456* |
|  | (0.209) | (0.190) | (0.534) | (0.112) | (0.375) | (0.255) | (0.173) | (0.131) | (0.291) | (0.129) | (0.417) | (0.248) |
| **PStability** | 0.761*** | 0.117 | -0.038 | 0.032 | -0.032 | -0.188 | -0.620*** | -0.060 | 0.270 | 0.149* | -0.814*** | -0.501*** |
|  | (0.130) | (0.118) | (0.350) | (0.075) | (0.239) | (0.168) | (0.109) | (0.083) | (0.184) | (0.081) | (0.263) | (0.156) |
| **GDP** | -1.069 | 0.561 | 3.014 | -1.558* | 5.588** | 1.701 | -3.038* | 0.455 | -1.614 | -3.033** | 20.36*** | 9.543*** |
|  | (1.451) | (1.318) | (3.808) | (0.807) | (2.632) | (1.826) | (1.679) | (1.285) | (2.811) | (1.245) | (4.068) | (2.404) |
| **INFLA** | -0.288 | 0.369 | 0.726 | -0.186 | 0.976 | 0.590 | -1.668*** | -0.235 | -0.184 | 0.213 | 2.469** | 1.160* |
|  | (0.428) | (0.388) | (1.100) | (0.233) | (0.782) | (0.528) | (0.450) | (0.346) | (0.759) | (0.337) | (1.097) | (0.647) |
| **Constant** | 1.115 | 6.802*** | 3.426 | 5.119*** | -1.476 | -2.643** | 1.471 | 3.984*** | 5.477*** | 3.062*** | -7.756*** | 0.526 |
|  | (0.835) | (0.758) | (2.135) | (0.393) | (1.315) | (0.906) | (0.757) | (0.576) | (1.270) | (0.543) | (1.773) | (1.046) |
| **Observations** | **486** | **486** | **486** | **486** | **486** | **486** | **468** | **468** | **468** | **468** | **468** | **468** |
| **Year Effects** | **Yes** | **Yes** | **Yes** | **Yes** | **Yes** | **Yes** | **Yes** | **Yes** | **Yes** | **Yes** | **Yes** | **Yes** |
| **Country Effects** | **Yes** | **Yes** | **Yes** | **Yes** | **Yes** | **Yes** | **Yes** | **Yes** | **Yes** | **Yes** | **Yes** | **Yes** |
| **R^2^** | **0.780** | **0.535** | **0.367** | **0.660** | **0.262** | **0.174** | **0.677** | **0.665** | **0.195** | **0.274** | **0.314** | **0.296** |
| **Wald Chi2** | **435***** | **387***** | **355***** | **278***** | **392***** | **483***** | **390***** | **419***** | **467***** | **397***** | **502***** | **304***** |
| **LM Statistics (p-value)** | **000** | **000** | **000** | **000** | **000** | **000** | **000** | **000** | **000** | **000** | **000** | **000** |
| **Sargan test  (p-value)** | **522** | **735** | **574** | **408** | **484** | **385** | **589** | **693** | **657** | **285** | **375** | **473** |

Note: This table presents the results for different ages of Banks’ Life Cycle, which are classified as mature banks (Panel A) and young banks (Panel B), across conventional banks in the MENA region. The table presents Three-Stage Least-Squares (3SLS) estimations for the full sample of banks identifying the impact of terrorism risk on a bank’s financial stability, which is represented by bank risk as measured through the credit risk, liquidity risk and insolvency risk (Panel A), and bank performance as measured through profitability (ROAA and ROEA), COST/INCOME ratio (Panel B). Our estimated models are defined as:

${\mathbf{Performance}_{\mathbf{it}}\mathbf{=}{\boldsymbol{\beta}_{\mathbf{0}}}_{\mathbf{it}}\mathbf{+}\boldsymbol{\beta}_{\mathbf{1}}\mathbf{Terrorism risk}{}_{\mathbf{it}}\boldsymbol{+ \phi Control+Country effects+Year effects+}}\boldsymbol{\varepsilon}_{\mathbf{it}}$ **(1)**

${{\mathbf{Risk}_{\mathbf{it}}\mathbf{=}{\boldsymbol{\beta}_{\mathbf{0}}}_{\mathbf{it}}\mathbf{+}\boldsymbol{\beta}_{\mathbf{1}}\mathbf{Terrorism risk}_{\mathbf{it}}}\boldsymbol{+ \phi control +Contry effects+ Year effects+}}\boldsymbol{\varepsilon}_{\mathbf{it}}$ **(2)**

Models are tested for the period of nine years from 2010. P-values in parentheses, *p < 0.10, **p < 0.05, ***p < 0.01. LM and Sargan tests show that our models are correctly identified, and our selected IVs are valid. See Table 2 for variable definitions.

1. Life cycle is defined as the number of years since the establishment of the bank. [↑](#footnote-ref-1)
